# Structure and dynamics of the ASB9 CUL-RING E3 Ligase

**DOI:** 10.1038/s41467-020-16499-9

**Published:** 2020-06-08

**Authors:** Ryan J. Lumpkin, Richard W. Baker, Andres E. Leschziner, Elizabeth A. Komives

**Affiliations:** 1 Department of Chemistry and Biochemistry, University of California, San Diego, 9500 Gilman Drive, La Jolla, CA 92092-0378 USA; 2 Department of Cellular and Molecular Medicine, School of Medicine, University of California, San Diego, La Jolla, CA 92093 USA; 3 Section of Molecular Biology, Division of Biological Sciences, University of California, San Diego, La Jolla, CA 92093 USA; 4 Present Address: Department of Biochemistry and Biophysics, School of Medicine, University of North Carolina, Chapel Hill, Chapel Hill, NC USA; 5 Present Address: Lineberger Comprehensive Cancer Center, University of North Carolina, Chapel Hill, Chapel Hill, NC USA

**Keywords:** Cryoelectron microscopy, Supramolecular assembly

## Abstract

The Cullin 5 (CUL5) Ring E3 ligase uses adaptors Elongins B and C (ELOB/C) to bind different SOCS-box-containing substrate receptors, determining the substrate specificity of the ligase. The 18-member ankyrin and SOCS box (ASB) family is the largest substrate receptor family. Here we report cryo-EM data for the substrate, creatine kinase (CKB) bound to ASB9-ELOB/C, and for full-length CUL5 bound to the RING protein, RBX2, which binds various E2s. To date, no full structures are available either for a substrate-bound ASB nor for CUL5. Hydrogen–deuterium exchange (HDX-MS) mapped onto a full structural model of the ligase revealed long-range allostery extending from the substrate through CUL5. We propose a revised allosteric mechanism for how CUL-E3 ligases function. ASB9 and CUL5 behave as rigid rods, connected through a hinge provided by ELOB/C transmitting long-range allosteric crosstalk from the substrate through CUL5 to the RBX2 flexible linker.

## Abstract (web summary)

Multi-subunit Cullin (CUL)-RING ligases (CRL) form the largest family of E3 ligases and are composed of a substrate receptor, a CUL, and a RING-box (RBX) protein. Here, the authors use cryo-EM and HDX-MS to characterise the ASB9 CUL-RING E3 ligase and present the structure of ASB9-ELOB/C bound to the substrate creatine kinase and the full-length CUL5 structure in complex with RBX2, and they propose a revised allosteric mechanism for CUL-E3 ligase function.

## Introduction

Ubiquitylation is a post-translational protein modification in which the 8.5 kDa protein Ubiquitin (Ub) is covalently attached to a substrate protein following activation by the Ubiquitin-activating enzyme (E1) and transfer to a Ubiquitin-conjugating enzyme (E2). Ubiquitin ligases (E3) facilitate the highly-specific covalent attachment of activated Ubiquitin (Ub) to substrate proteins through an isopeptide bond on an exposed lysine residue. Ubiquitylation leads to two general classes of modification, mono- and poly-ubiquitylation, wherein a molecule of Ubiquitin is linked to one of the seven lysines in another molecule of Ubiquitin. K48 polyubiquitin chains are the most studied form of polyubiquitylation and are responsible for targeting substrate proteins for proteasomal degradation^1^.

The multi-subunit Cullin (CUL)-RING ligase (CRL) is the largest family of E3 ligases, and it is responsible for up to 20% of degradation through the proteasome^2^. CRLs share a common structure, composed of a substrate receptor(s), a CUL, and a RING-box (RBX) protein. The RBX subunit is responsible for recruiting Ubiquitin-charged E2 enzymes to the ligase^1^. Human proteins CUL2 and CUL5 bind substrate receptors through the adaptor proteins Elongin B (ELOB) and Elongin C (ELOC). The vHL-box and SOCS-box motifs in CUL2 and CUL5 cause them to recruit vHL-like or SOCS-box-containing substrate receptors, respectively. Several protein families contain SOCS-box domains, including the canonical Suppressor of Cytokine Signaling (SOCS), Single Stranded Binding (SSB), WD40 SOCS-box (WSB), and Ankyrin Repeat and SOCS-box (ASB) families^3^.

The 18 ASB proteins comprise the largest family of SOCS-box domain-containing E3 ligase substrate receptors. Each ASB protein contains an ankyrin repeat domain and a SOCS-box domain with CUL5-box and BC box motifs, and they specifically associate with CUL5 through the Elongin B and C (ELOB/C) adapter proteins^4–6^. A few ASB proteins have known substrates that are marked for degradation: ASB2-CRL ubiquitylates Filamins A and B, ASB3 and 4-CRLs ubiquitylate tumor necrosis factor receptor II, and ASB11-CRL ubiquitylates Notch ligand DeltaA^7^.

Creatine Kinase brain-type (CKB) and mitochondrial type (MtCK) have been shown to be degraded in vivo in an ASB9-dependent manner^8,9^. ASB9 binds a dimer of CKB with sub-nanomolar affinity^10^ with residues 19–24 being especially important for high affinity binding^10,11^. Structures of ASB9 lacking the SOCS box domain^12^, and including the SOCS-box bound to ELOB and ELOC have been determined^7^. In addition, structures of SOCS2-ELOB/C bound to the N-terminal half of CUL5 (PDB 4JGH)^13^ and the C-terminal half of CUL5 bound to RBX1 (3DPL)^14^ have been determined. While some details have been shown in a fragmented manner, a full mechanistic understanding of the ASB9 CRL assembly, substrate recognition, and dynamics remain unknown.

We present a full model of the substrate-bound ASB9-ELOB/C-CUL5-RBX2 E3-ligase complex based on cryo-electron microscopy (cryo-EM) structures of CKB-ASB9-ELOB/C and of CUL5. By combining cryo-EM, hydrogen–deuterium exchange (HDX), and homology modeling, we define the general architecture of the complex and propose conformational dynamics that underlie substrate recognition and Ubiquitin loading. Hydrogen–deuterium exchange-mass spectrometry (HDX-MS) data confirms binding sites between CKB-ASB9 and ELOB/C-CUL5. Most importantly, the HDX-MS reveals a pathway of allostery that connects substrate binding to conformational changes near important modules in CUL5 that control ligase activity, including the site of CUL5 neddylation and RBX2-E2 binding. This allosteric pathway suggests that substrate binding itself serves a mechanistic role in the activity of CUL-RING ligases.

## Results

### Structure of the CKB-ASB9-ELOB/C complex by Cryo-EM

**Fig. 1:**
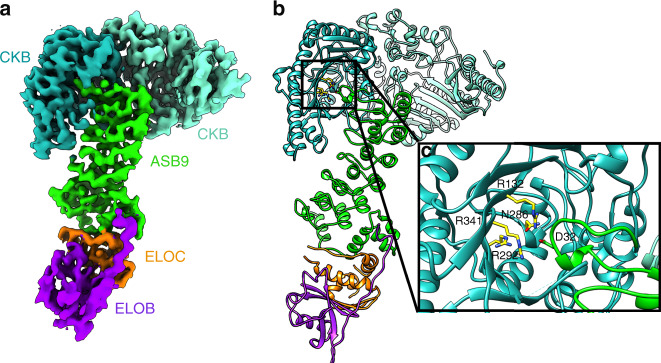
Structure of the CKB substrate bound to the ASB9 substrate receptor and adapter proteins ELOB/ELOC. **a** Cryo-EM density of CKB-ASB9-ELOB/C, colored by subunit, with a final overall resolution of 4.1 Å. **b** Molecular model for CKB-ASB9-ELOB/C complex. The model was built by fitting crystal structures (CKB: 3DRB; ASB9/ELOB/C: 3ZKJ), de novo building of small regions using RosettaCM, and overall refinement using Rosetta Relax. **c** The ASB9 N-terminus inserts as one helical turn into the active site of one CKB monomer positioning the ASB9 aspartate D32, which was shown to be critical for high affinity binding, in a basic pocket (R132, N286, R292, R341).

To study the assembly of the full CUL5 complex, we prepared a number of different sub-complexes of the E3 ligase complex containing CKB-ASB9-ELOB/C-CUL5-RBX2 (Supplementary Fig. 1). We previously determined that the *K*_D_ for the CKB-ASB9 interaction is in the picomolar range^10^. Initial experiments preparing sub-complexes revealed that we could prepare CKB-ASB9-ELOB/C and mix that with ELOB/C-CUL5-RBX2 to form the full ligase containing only one ELOB/C subunit. These experiments therefore revealed that ELOB/C could be exchanged whereas the CKB-ASB9 interaction and the CUL5-RBX2 interaction were essentially irreversible. Attempts to determine a cryo-EM structure of the full complex were unsuccessful due to particle disintegration at the air–water interface. To overcome this obstacle, we determined cryo-EM structures of two subcomplexes and modeled the full complex using our cryo-EM data and previously determined crystal structures. We first determined a 4.1 Å structure of the substrate–receptor complex containing CKB, ASB9, ELOB, and ELOC (Fig. 1a and Supplementary Fig. 2). A model was refined from this cryo-EM map using crystal structures of the CKB homodimer (PDB 3DRB) and the ASB9-ELOB/C heterotrimer (PDB 3ZKJ) (Fig. 1b and Supplementary Fig. 2). CKB binds as a dimer to the N-terminal ankyrin repeat of ASB9 as previously predicted from HDX-MS data, kinetic data, and modeling^10,11^. The structure of the CKB homodimer (PDB 3DRB) has one monomer in an “open” conformation and the other in a “closed” conformation. The model that best fit the cryo-EM density was that with both CKB monomers in the “open” conformation.

ASB9 from ASB9-ELOB/C (3ZNG) aligned closely with the Cryo-EM density for the ASB9 subunit. The structure of ASB9 residues 25–34 was resolved to reveal a partial fold of a helix and a turn inside the active site of one monomer of the CKB dimer (Fig. 1c). Residues 19–34 of the N-terminus of ASB9 were present in the structure of the apo-ASB9-2 splice variant (3D9H)^12^ and residues 23–34 were modeled for the docked structure of CKB and ASB9^11^, but neither of these predictions matched the cryo-EM density. The partial helix positions D32 in the active site of one CKB monomer (Fig. 1c), consistent with previous biophysical experiments demonstrating the critical role of this residue in the interaction^11^. Data collection, refinement, and model building statistics are listed in Table. 1.

**Table 1:** Cryo-EM data collection, refinement, and validation statistics.

	Asb9-CKB-EloB-EloC (PDB: 6VPH, EMD-21120)	Cullin 5-RBX2 (PDB: 6VPI, EMD-21121)
Data collection		
Microscope	Talos Arctica	Talos Arctica
Camera	K2 Summit	K2 Summit
Camera mode	Counting	Counting
Magnification	36,000	36,000
Voltage (kV)	200	200
Total electron exposure (e^−^/Å^2^)	50	60
Exposure rate (e^−^/pixel/s)	6.7	5.5
Defocus range (μm)	0.6–2.5	0.6–2.5
Pixel size (Å/pixel)	1.16	1.16
Micrographs collected (no.)	1983	591
Micrographs used (no.)	1735	316
Reconstruction		
3D processing package	cryoSPARC v2	cryoSPARC v2
Total extracted picks (no.)	1,309,964	415,601
Refined particles (no.)	368,831	44,372
Final particles (no.)	285,105	44,372
Symmetry	C1	C1
Resolution (global) (Å)	4.1	5.2
FSC 0.143 (unmasked/masked)	4.9/4.1	
Local resolution range (Å)	3.5–8.0	
Map sharpening *B-*factor	−232	
Refinement		
Model refinement package	Rosetta	Rosetta
Number of models	10	1
Nonhydrogen atoms	9329 (per model)	
Protein residues	1185 (per model)	814
* B* factors (Å^2^)		
Protein residues	87.42	–
RMS deviations		
Bond lengths (Å)	0.0139	–
Bond angles (°)	1.35	–
Validation		
MolProbity score	1.17	2.09
ClashScore	1.62	13.62
Poor rotamers (%)	0	–
CaBLAM outliers (%)	0	–
Ramachandran plot		
Favored (%)	96.14	–
Allowed (%)	3.43	–
Disallowed (%)	0.43	–
EMRinger score	1.4	N/A
Map CC (*CCmask*)	0.752	0.552

### Structure of full-length CUL5-RBX2

**Fig. 2:**
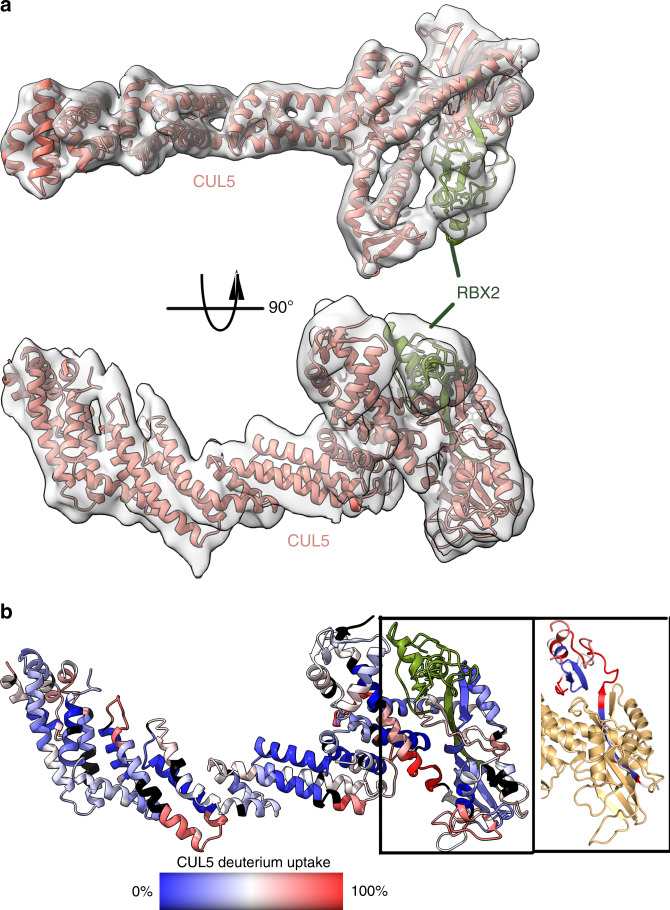
Structure of full-length CUL5-RBX2 complex. **a** Cryo-EM map of CUL5-RBX2 shown with the molecular model of CUL5 bound to RBX2. Model was built from crystal structures of CUL5_NTD_ (4JGH) and CUL5_CTD_ + RBX1 (3DPL). **b** Model of CUL5-RBX2 showing the amide exchange results from the HDX-MS data. The exchange after 2 min varied from 0% (blue) to 100% (red). Regions that were not covered in the HDX-MS data are colored black. **c** The amide exchange after 2 min for RBX2 is displayed on the same color scale as for CUL5.

To date, no full-length structure of CUL5 is available, however a structure of SOCS2-ELOB/C-CUL5_NTD_ (4JGH) and of CUL5_CTD_-RBX1 (3DPL) are available. We determined a ~6 Å cryo-EM structure for the full-length CUL5-RBX2 complex (Fig. 2a and Supplementary Fig. 3). A strong preferred orientation in the cryo-EM grid limited the resolution of our map, but we were able to partially overcome this orientation bias by collecting data with a 20° tilt. Despite the moderate resolution of our cryo-EM map, both CUL5 crystal structures could be unambiguously docked into the cryo-EM density (Supplementary Fig. 3). Using these crystal structures as a starting point for modeling, we built a complete model of the CUL5-RBX2 complex (Fig. 2a and Supplementary Fig. 3). Data collection, refinement, and model building statistics are listed in Table 1.

### Full model of the ASB9-CRL

**Fig. 3:**
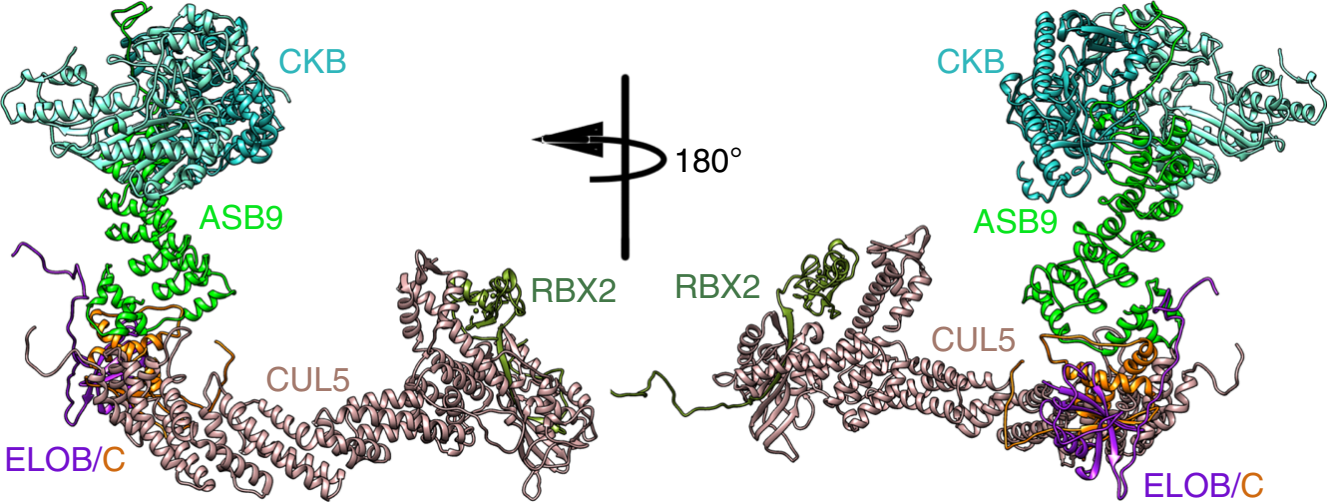
Model of the full ASB9-CRL. A complete model of the un-activated ASB9-CRL was assembled from the cryo-EM model of CKB-ASB9-ELOB/C and the cryo-EM model of CUL5. The colors are as follows: CKB dimer (light sea green, aquamarine), ASB9 (green), ELOB/C (orange/purple), RBX2 (olive).

The structural model of the CKB-ASB9-ELOB/C was combined with the model of CUL5-RBX2 by superposition of ELOB/C. This process yielded a model of the ASB9CRL with the substrate, CKB bound (Fig. 3). This model captures the cryo-EM structure of the substrate bound to ASB9, and the full structure of CUL5 bound to RBX2.

### HDX-MS mapping of protein–protein interfaces in the CKB-bound ASB9-CRL

HDX-MS is useful for determining the exposure of backbone amides in proteins. We previously showed that HDX-MS measurement of differences in the number of rapidly exchanging amides is useful for revealing unfolded or intrinsically disordered regions^15^, protein–protein interfaces^16^, and sites of allostery^17^. To complement the cryo-EM data on full-length CUL5-RBX2, we analyzed this sub-complex by HDX-MS. Interestingly, throughout the entire core of CUL5, the amide exchange was extremely low, indicating that the three helical sub-domains form one contiguous well-folded structure (Fig. 2b and Supplementary Figs. 4 and 5A). In contrast to the low exchange throughout CUL5, RBX2 residues 41–47, 52–58, and 62–73 exchange over 80% strongly suggesting that RBX2 has a flexible tether that could adopt many conformations bringing it closer to the substrate (Fig. 2c and Supplementary Fig. 5B).

**Fig. 4:**
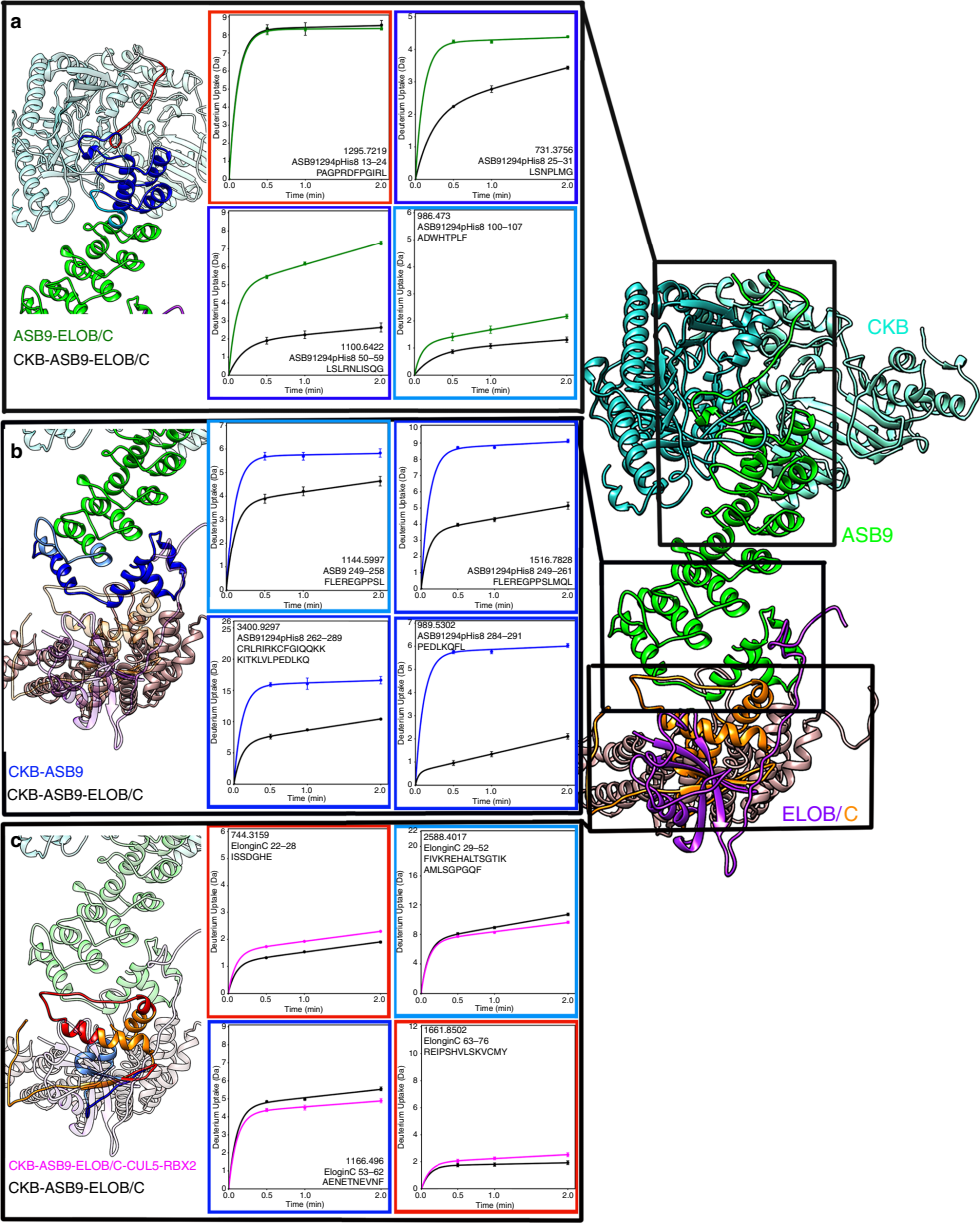
HDX-MS reveals protein interaction surfaces. **a** The HDX-MS data showed that ASB9 (green circles) residues 13–24 exchanged completely in both free ASB9 and in CKB-bound ASB9 (black circles) suggesting this region does not interact with CKB. ASB9 residues 25–99 markedly exchange upon interaction with CKB and protection extended through residue 107. **b** The ASB9 (blue circles) SOCS box markedly decreased exchange upon binding to ELOB/C (black circles). **c** ELOC (black circles) showed both increased (residues 22–28 and 76–101) and decreased (residues 29–52, 53–62) exchange in the presence of CUL5 (magenta circles) suggesting a hinging motion. For all HDX-MS data, at least two biological replicates were analyzed each with three technical replicates. Data are represented as mean values ± SEM of three technical replicates due to processing software limitations, however the LEAP robot provides highly reproducible data for biological replicates and therefore error bars are sometimes within the symbols. Source data for the HDX-MS results, (**a**)–(**c**), are provided as Source Data files.

We next performed HDX-MS studies on various complexes to gain information about the interfaces between the individual proteins^16^. Differences in deuterium exchange of the N-terminal ankyrin repeat of ASB9 between ASB9-ELOB/C and CKB-ASB9-ELOB/C reflect the impact of association of CKB with ASB9. Previous HDX-MS experiments identified that residues 25–42 of ASB9 were protected by CKB but did not go into greater detail^10^. In the current study, peptides covering ASB9 residues 1–8 were not detected, but residues 13–24 were found to be maximally exchanging in all states (Fig. 4a and Supplementary Fig. 6). Peptides corresponding to ASB9 residues 25–31, 25–42, 43–52, 50–59, 64–84, 85–99, and 100–107 (Fig. 4a and Supplementary Fig. 7A) all showed statistically significant decreases in exchange upon association with CKB, demonstrating that at least two ankyrin repeats of ASB9 engage in the CKB binding interaction. The decreased exchange was most notable in residues 50–59, the second helix of the first ankyrin repeat, with five fewer amides exchanging in the presence of CKB.

Differences in deuterium uptake for CKB-ASB9 and CKB-ASB9-ELOB/C reflect the effects of ELOB/C interaction with the SOCS-box of ASB9. Peptides corresponding to ASB9 residues 176–214, 218–237, 238–248, 249–258, 249–261, 262–289, and 284–291 all showed decreased exchange in the presence of ELOB/C (Fig. 4b and Supplementary Fig. 7B). We analyzed the two overlapping peptides corresponding to residues 249–258 and residues 249–261, and were able to determine that the majority of the difference in uptake was due to decreased exchange in residues 259–261^18^ (Fig. 4b). These residues exchanged in ASB9 alone whereas less than one amide exchanged in the ELOB/C-bound state. This region corresponds to the two SOCS box subdomains; the BC-box motif, L-X-X-L-C-R, residues 258–263 in ASB9 and the CUL5-box motif is L-X-Φ-P-X-X-Φ-X-X-Ω-L, residues 281–291.

By comparing the deuterium exchange in ASB9-ELOB/C with that in ASB9-ELOB/C-CUL5-RBX2, we discovered that the conformation of ELOC is altered upon binding to CUL5 (Fig. 4c). ELOC residues 53–62 showed decreased exchange in the presence of CUL5 consistent with a binding interaction. Remarkably, regions on either side of residues 53–62, residues 22–28, and residues 76–101, showed increased exchange in the presence of CUL5 (Fig. 4c and Supplementary Figs. 7C and 8). These results suggested a rearrangement and/or hinging at ELOC takes place upon binding of CUL5.

### HDX-MS reveals crosstalk between non-adjacent subunits of the ASB9-CRL

By comparing variations of ASB9-CRL complexes, we discovered long-range changes in amide exchange indicative of allosteric crosstalk between non-adjacent subunits. We first compared ASB9-ELOB/C-CUL5-RBX2 with and without neddylation of CUL5. While we thought neddylation might induce changes in the substrate receptor, none were observed.

**Fig. 5:**
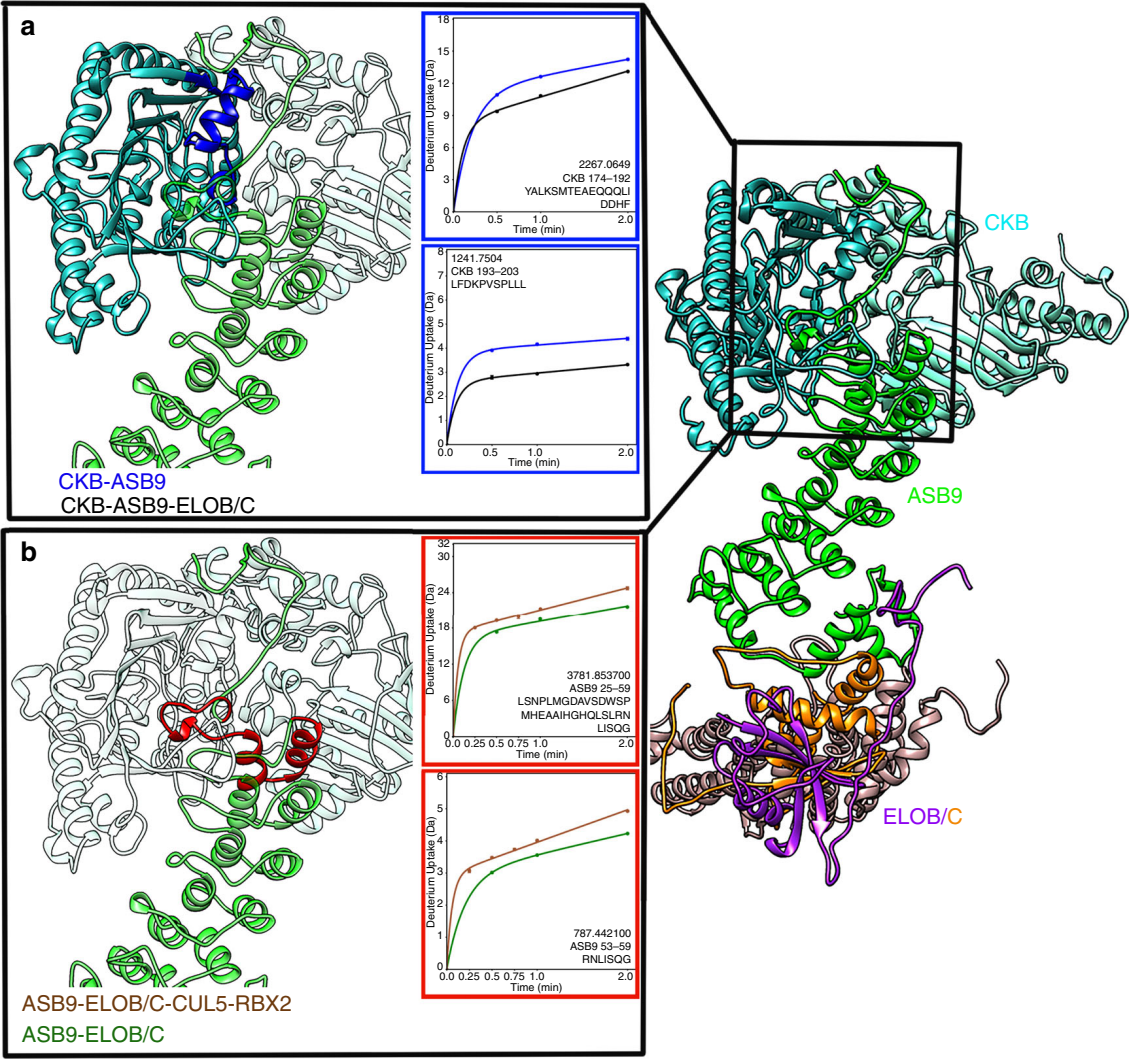
Long-range allosteric crosstalk was observed by HDX-MS. **a** CKB (blue circles) residues 174–203 (bound at the N-terminus of ASB9) upon binding of ELOB/C (black circles) to the C-terminal end of ASB9. **b** The first ankyrin repeat of ASB9 (green circles) showed increased exchange when CUL5 was present (brown circles). For all HDX-MS data, at least two biological replicates were analyzed each with three technical replicates. Data are represented as mean values ± SEM of three technical replicates due to processing software limitations, however the LEAP robot provides highly reproducible data for biological replicates and therefore error bars are sometimes within the symbols. Source data for the HDX-MS results, (**a**) and (**b**), are provided as Source Data files.

The amide exchange into CKB-ASB9-ELOB/C complex was compared to that of the CKB-ASB9 complex, to observe any differences in the substrate (CKB) and substrate receptor (ASB9) when ELOB/C was present. Indeed, CKB residues 177–193 and 193–203 showed decreased exchange in the presence of ELOB/C (Fig. 5a and Supplementary Fig. 9). This result indicates long-range communication through the substrate receptor, ASB9, to the substrate, CKB, when the ASB9 complex engages ELOB/C. We also analyzed how the ligase responded to a lack of substrate by comparing deuterium exchange into the ASB9-ELOB/C complex to exchange into the ASB9-ELOB/C-CUL5-RBX2 complex. In the absence of CKB, ASB9 residues 25–42 and 53–59 showed higher deuterium exchange in the ASB9-ELOB/C-CUL5-RBX2 complex as compared to that in the ASB9-ELOB/C complex (Fig. 5b). These results reveal even longer-range communication through the ASB9 substrate receptor and the ELOB/C adapter proteins so that when ELOB/C engage CUL5 in the absence of the substrate, the substrate binding site on ASB9 is more dynamic.

**Fig. 6:**
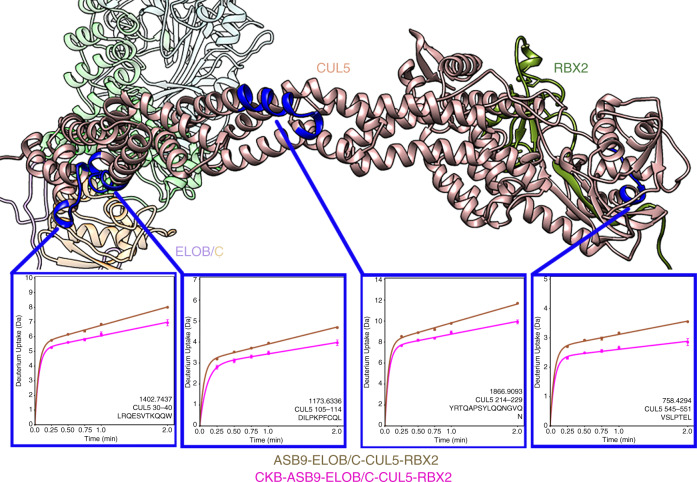
CKB binding induces changes in HDX in CUL5. CUL5 (brown circles) residues 30–40, 105–114, 214–229, and 545–551 showed decreased exchange upon CKB binding (magenta circles) to the ASB9 CRL. For all HDX-MS data, at least two biological replicates were analyzed each with three technical replicates. Data are represented as mean values ± SEM of three technical replicates due to processing software limitations, however the LEAP robot provides highly reproducible data for biological replicates and therefore error bars are sometimes within the symbols. Source data for the HDX-MS results are provided as Source Data files.

The most remarkable example of the observed long-range allostery was how CUL5 appeared to respond to the presence of substrate, CKB. When we compared the ASB9-ELOB/C-CUL5-RBX2 complex to the CKB-ASB9-ELOB/C-CUL5-RBX2 complex, decreased exchange in several regions of CUL5 was observed. This included CUL5 residues 7–21, 30–40, 105–114, 161–176, 214–229, 269–278, and 545–551. This result is most consistent with a twisting of the CUL5 molecule upon binding of CKB to ASB9 (Fig. 6 and Supplementary Fig. 10).

## Discussion

We were able to obtain a 4.1 Å cryo-EM structure of the ASB9-CKB complex, revealing the structure of an ASB substrate receptor bound to a *bona fide* ASB-CRL substrate. The cryo-EM density revealed an unexpected structuring of ASB9 residues 25–34, which form a partial helix in the active site of one subunit of the CKB dimer positioning Asp 32, a residue previously identified as critical for binding affinity^10,11^. ASB9 residues 13–24 exchanged over 90% in both the absence and presence of CKB whereas residues 25–31 showed significant protection upon CKB binding consistent with the structure formation observed by cryo-EM. These data suggest that the structure of ASB9 residues 25–34 may position D32 in the CKB active site leading to the higher affinity binding of ASB9 1–252 as compared to the weaker binding of ASB9 35–252 as reported previously^10^. It is interesting to speculate that the disordered N-terminal 35 residues of ASB9 may provide the promiscuous interactions necessary for interaction with other putative ASB9 substrates which so far have only been identified by affinity mass spectrometry^6^. The list of putative ASB9 substrates includes many metabolic enzymes, which, like CKB, adopt dimeric structures.

We were also able to obtain a cryo-EM structure of full-length CUL5 bound to RBX2, which has previously been only structurally characterized using fragments of the N- and C-terminal domains of CUL5. HDX-MS data of CUL5 showed low exchange throughout the core of the entire CUL5 with only the connecting loops between the helices showing higher exchange. The core of CUL5 exchanged very little throughout, indicating that the three repeated helical bundles form one folded structure without any obvious flexibility throughout the helical repeats. In fact, engagement of the CUL5 with CKB-ASB9-ELOB/C actually resulted in even further decreases in exchange throughout the CUL5 molecule again suggesting that in the full E3 ligase, the CUL5 behaves as a rigid rod.

Comparison of the HDX-MS data for the ASB9-ELOB/C vs. ASB9-ELOB/C-CUL5-RBX2 revealed that the presence of CUL5-RBX2 causes increased exchange in the first ankyrin repeat of ASB9. Increased exchange is generally considered indicative of increased dynamics. It is interesting to speculate that increased dynamics at the substrate binding site may promote CKB binding by a fly-casting mechanism^19^. Assembly of the CRL without its substrate bound would be dangerous as CRL components are known to autoubiquitylate^20^. A mechanism such as fly-casting^19^ to promote substrate binding under these conditions might be desirable. Comparison of the HDX-MS data for the CKB-ASB9 vs. CKB-ASB9-ELOB/C revealed the converse allosteric changes. In this case, the regions of the CKB homodimer that interface with ASB9 showed decreased exchange in the presence of ELOB/C. Finally, comparison of the HDX-MS data for the CKB-ASB9-ELOB/C-CUL5-RBX2 vs. the ASB9-ELOB/C-CUL5-RBX2 complex showed that CKB binding to ASB9 induced decreased exchange at specific surface loops in CUL5 spanning from residue 25 through residue 551. This may possibly be due to CKB binding inducing changes in ELOB/C which then cause twisting of the CUL5_._ These results imply that CKB may induce allosteric crosstalk through ASB9-ELOB/C and then through CUL5, a series of three other proteins.

**Fig. 7:**
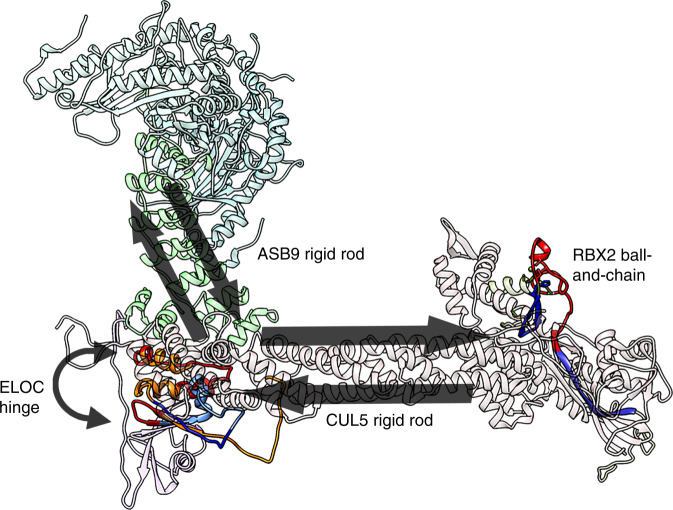
The predicted allosteric path from substrate, CKB, through the CRL. CKB binding communicates through the substrate receptor, ASB9 which behaves as a rigid rod, through ELOB/C which behaves as a hinge, and finally through CUL5, which also behaves as a rigid rod. Both ASB9 and CUL5 appear to move as rigid bodies in the allosteric network. The network terminates in RBX2, which appears to be connected to CUL5 by a flexible (very high amide exchange) tether.

The mechanism of allostery in the ASB9 CRL appears to involve ASB9 and CUL5, which behave as rigid rods, connected by a hinge, ELOC. The amide exchange throughout the folded core of ASB9 and CUL5 is very low in all sub-complexes that were studied. ASB9 is an ankyrin repeat protein which is stabilized by interactions between its ankyrin repeats. As was observed in IκBα^21^, such a one-dimensional architecture is an ideal structure through which to transmit allosteric changes since it is minimally frustrated^22^ through its long cylindrical core and should move as a rigid body. It is interesting that a similar phenomenon is observed in CUL5, which could be considered a quasi-repeat protein, as it has a similar cylindrical architecture of repeated helical bundles, and it also appears to move as a rigid body. Neither the HDX-MS data nor the cryo-EM data show any indication of flexibility within CUL5 as had been previously suggested by simulations^11,23^. In fact, upon substrate binding three discontinuous sites in CUL5 show decreased exchange as if the entire helical bundle appears to become more rigid and less dynamic (Fig. 6). Connection between the substrate binding site on ASB9 and RBX2 bound at the C-terminal end of CUL5 is provided by ELOC. The HDX-MS data showed decreased exchange in one region of ELOC and increased exchange in the segments preceding and following this segment. When viewed on the structure, this protection/deprotection appears to suggest a hinging motion in which ELOC may allow the right angle between ASB9 and CUL5 to become more acute (Fig. 7). We did not think it appropriate to attempt to model this movement as such modeling will require extensive molecular dynamics simulations. Ubiquitin transfer may also be facilitated by RBX2. Our HDX-MS data reveal that the strand connecting RBX2 to CUL5 is completely exchanging suggesting it maybe behave like a flexible tether, enabling Ub transfer by a ball-and-chain mechanism. The RBX2 tether may not reach the substrate to transfer the first Ub, but may add additional Ubs after a Ring between Ring ligase installs the first Ub on bound substrates as has been recently shown for CUL5 which requires ARIH2^24^.

## Methods

### Expression vectors

Human ASB9-1 in pNIC-CTHF was obtained from the Structural Genomics Consortium and subcloned into pHis8 (Kan^R^) with an N-terminal 8×His tag and Human CKB was subcloned into pET11a (Amp^R^) as previously described^10^. Human ELOB (full-length) and ELOC (17-112) were obtained in pACYC (Cam^R^) (Structural Genomics Consortium). Human CUL5 and mouse RBX2 were obtained in pRSFDuet (Kan^R^) with an N-terminal His tag, TEV cleavage site, and GB1 tag on CUL5 (gift from Nevan Krogan). 6×His-GB1-TEV-CUL5 was subcloned into pET28a (Kan^R^). All of these subcloning steps were performed by restriction digestion and ligation of the resulting fragments. RBX2 was subcloned into pET11a (Amp^R^). PCR Primer sequences for subcloning of RBX2 are contained in Supplementary Table 1. All constructed plasmids were sequenced for verification.

### Protein expression

ASB9 was co-expressed with ELOB/C and/or CKB using sequential transformation with the vectors described above into BL21(DE3) *Escherichia coli* cells (New England Biolabs) for ASB9-CKB, ASB9-ELOB-ELOC, and ASB9-CKB-ELOB-ELOC co-expression. Vectors containing ELOB/C, CKB, or both were transformed into competent BL21(DE3) cells after which those cells were made competent again. ELOB/C in pACYC was selected by Chloramphenicol (CAM) resistance, and CKB in pET11a was selected by Ampicillin (AMP) resistance. The vector for expression of ASB9 was transformed into ELOB/C + CKB-containing BL21(DE3) cells and plated on a Kanamycin (KAN)-CAM-AMP LB agar plate. The pET28a vector for expression of CUL5 and the pET11a vector for expression of RBX2 were co-transformed into ELOB/C-containing BL21(DE3) cells and plated on a KAN-CAM-AMP LB agar plate for co-expression of CUL5/RBX2/ELOB/C.

All proteins were expressed as follows. A 5 mL M9-ZN (1.5× M9 salts, NZ-Amine media, 0.8% Dextrose, 1 mM MgSO_4_, 0.2 mM CaCl_2_) overnight culture was inoculated with a single colony from the plate. A 20 mL M9-ZN starter culture was inoculated with 2 mL of the overnight culture and grown for 3 h at 37 °C. The 1 L M9-ZN growth culture was inoculated with the entire 20 mL starter culture and grown until OD_600_ = 0.8. After placing the cultures on ice for 15 min, protein expression was induced by addition of IPTG to a final concentration of 0.5 mM, and the cultures were transferred to an 18 °C incubator for 16–18 h. Because RBX2 contains a Zn binding domain, the RBX2-containing cultures were brought to 200 μM Zn by the addition of a 1 M solution of ZnCl_2_ just prior to induction.

### Protein purification

Cells from 1 L of culture were pelleted by centrifugation at 3000 × *g* for 10 min, then re-suspended in 40 mL resuspension buffer (50 mM Tris–HCl, pH 8.0, 100 mM NaCl, 10 mM imidazole, pH 8.0, 2 mM β-mercaptoethanol, 5% glycerol) with Sigma Protease Inhibitor Cocktail (P2714) and 5 mM PMSF. Cells were lysed on ice by sonication with ten 30 s pulses with 45 s cool-down between each pulse. The lysate was clarified by centrifugation at 20,000×*g* for 45 min. The clarified lysate was incubated with 2 mL Ni-NTA (in resuspension buffer) for 2 h at 4 °C with rocking. Ni-NTA beads were pelleted by centrifugation at 700 × *g* for 5 min. The supernatant was discarded, and the beads were washed with 10 mL wash buffer (50 mM Tris–HCl, pH 8.0, 100 mM NaCl, 25 mM imidazole, pH 8.0, 2 mM β-mercaptoethanol, 5% glycerol) for 30 min at 4 °C. Ni-NTA beads were again pelleted by centrifugation at 700 × *g* for 5 min. The supernatant was discarded, and the beads were washed with 10 mL elution buffer (50 mM Tris–HCl, pH 8.0, 100 mM NaCl, 250 mM imidazole, pH 8.0, 2 mM β-mercaptoethanol, 5% glycerol) for 30 min at 4 °C. Ni-NTA beads were again pelleted by centrifugation at 700 × *g* for 5 min. The supernatant was transferred to a 12–14 kDa Dialysis bag and dialyzed overnight in dialysis buffer (20 mM Tris–HCl, pH 8.0, 100 mM NaCl, 5% glycerol, 1 mM DTT). Samples were concentrated to 2 mL and purified using size-exclusion chromatography over a Superdex S200 16 × 600 column in dialysis buffer. Peak fractions were combined and concentrated to 5 µM for analysis by HDX-MS or 20 µM for structural determination by Cryo-EM.

Sample compositions were identified and characterized according to the presence of the desired proteins as assessed by size exclusion chromatography, sodium dodecyl sulfate-polyacrylamide gel electrophoresis (10–15% acrylamide gels), and nanospray liquid chromatography tandem-mass spectrometry on a Lumos mass spectrometer after trypsin digestion.

To prepare CKB-ASB9-ELOB/C or CUL5-RBX2 for structure determination, all proteins were co-expressed and purified as described above. Following size-exclusion by Superdex S200 16  ×  600, the samples were concentrated to 500 µL and re-purified on a S200 Increase 10/300 column. Collected fractions were concentrated to 20 μM.

CKB-ASB9, ASB9-ELOB/C, CKB-ASB9-ELOB/C, ASB9-ELOB/C-CUL5-RBX2, CKB-ASB9-ELOB/C-CUL5-RBX2, and ELOB/C-CUL5-RBX2, complexes were individually prepared for analysis by HDX-MS. CKB-ASB, ASB9-ELOB-ELOC, CKB-ASB9-ELOB/C, and ELOB/C-CUL5-RBX2 complexes were expressed and purified from multiple-expression systems as described above. ASB9-ELOB/C-CUL5-RBX2 and CKB-ASB9-ELOB/C-CUL5-RBX2 were formed by combining the lysates of ASB9-ELOB/C or CKB-ASB9-ELOB/C with ELOB/C-CUL5-RBX2 and purifying the combined proteins as described above.

### Hydrogen–deuterium exchange

HDX-MS experiments were conducted using a Waters nanoACQUITY UPLC system equipped with H/DX technology and a LEAP H/D-X PAL liquid handling system^25^. The H_2_O buffer was composed of 20 mM Tris–HCl, pH 8.0, 100 mM NaCl, 5% glycerol, 1 mM DTT, and 0.5 mM EDTA, matching the size-exclusion buffer used in the final stage of purification for each protein sample. This buffer was lyophilized and resuspended in D_2_O (D_2_O buffer). Using the LEAP robot, a 4 μL portion of a 5 μM protein sample was incubated for 5 min at 25 °C and then mixed with 56 μL of H_2_O buffer as a control or D_2_O buffer for deuteration times of 15, 30, 45, 60, or 120 s. The reaction was quenched with 60 μL of quench buffer (3 M guanidine, 0.1% formic acid, pH 2.66) at 0 °C. A portion of the quenched sample (50 μL) was injected into the sample loop and subsequently digested on an in-line pepsin column (Immobilized Pepsin, Pierce Inc.) at 15 °C. The digested samples were transferred to a BEH C18 Vanguard pre-column at 0 °C and subsequently separated by analytical chromatography (Acquity UPLC BEH C18, 1.7 μM, 1.0  ×  50 mm, Waters Corporation) using a 7–85% acetonitrile in 0.1% formic acid over 7.5 min also at 0 °C, and electrosprayed into the Waters SYNAPT G2Si quadrupole time-of-flight mass spectrometer. The mass spectrometer was set to collect data in the Mobility, ESI+ mode; mass acquisition range of 200–2000 (*m*/*z*); scan time 0.4 s. Continuous lock mass correction was accomplished with infusion of leu-enkephalin (*m*/*z* = 556.277) every 30 s (mass accuracy of 1 ppm for calibration standard). For peptide identification, the mass spectrometer was set to collect data in MSE, ESI+ mode instead.

The peptides were identified from triplicate MS^E^ analyses of 5 μM CKB-ASB9, ASB9-ELOB/C, and ELOB/C-CUL5-RBX2 samples with data analysis using PLGS 2.5 (Waters Corporation). Peptide masses were identified using a minimum number of 250 ion counts for low energy peptides and 50 ion counts for their fragment ions. The peptides identified in PLGS were then analyzed in DynamX 3.0 (Waters Corporation) using a cut-off score of 6.5, error tolerance of 5 ppm, and requiring that the peptide be present in at least 2 of the 3 identification runs. The peptides reported on the coverage maps are actually those from which data were obtained. The relative deuterium uptake for each peptide was calculated by comparing the centroids of the mass envelopes of the deuterated samples vs. the undeuterated controls^26^. For all HDX-MS data, at least two biological replicates were analyzed each with three technical replicates. Data are represented as mean values ± SEM of three technical replicates due to processing software limitations, however the LEAP robot provides highly reproducible data for biological replicates. The deuterium uptake was corrected for back-exchange using a global back exchange correction factor (typically 25%) determined from the average percent exchange measured in disordered termini of various proteins^27^. ANOVA analyses and *t*-tests with a *p*-value cutoff of 0.05 implemented in the program, DECA, were used to determine the significance of differences between HDX data points^18^. Deuterium uptake plots were generated in DECA (github.com/komiveslab/DECA) and the data are fitted with an exponential curve for ease of viewing. We do not report the exponential rates from these uptake curves because they are meaningless.

### Cryo-EM sample preparation and data collection

The CKB-ASB9-ELOB/C complex and the CUL5-RBX2 complex were purified as described above. The final protein buffer was 20 mM Tris, pH 7.5, 100 mM NaCl, 0.5 mM EDTA, 1 mM DTT. To increase the angular distribution of particles in our dataset, we supplemented protein samples with the detergent n-octyl-β-d-glucopyranoside (β-OG). All samples were prepared using UltraAuFoil R 1.2/1.3 300 mesh gold grids (Quantifoil GmbH) that were glow discharged for 30 s at 20 mAmp. Samples (4 μL) were applied to grids within 10 min of charging and blotting. Plunge freezing was performed using a Vitrobot Mark IV robot (Thermo Fisher). The robot was set to 100% humidity, 4 °C, blot force 20, and blot time 4 s.

Samples were imaged using a Talos Arctica TEM (Thermo Fisher) operating at 200 keV in nano probe mode at a magnification of 36,000×, with a final pixel size of 1.16 Å. Parallel illumination of the microscope was performed according to ref. ^28^. Dose fractionated movies were collected using a K2 Summit Direct Electron Detector (Thermo Fisher) operated in counting mode, with a defocus range of −0.6 and −2.5 µm, an exposure rate of ~6.7 e^−^/pixel/s, 200 ms frames, and a total exposure of ~50 e^−^/Å^2^. New camera gain references were collected before each dataset and the hardware dark reference was updated daily. Holes containing thin ice were manually selected and movies were collected automatically using the Leginon software suite (version 3.3)^29^. Motion correction and gain correction were performed using MotionCor2 (version 1.2.1)^30^, CTF estimation was performed on non-dose-weighted micrographs using CTFFIND4 version 4.1.2^31^, and particle picking was performed using DogPicker.py (within Appion version 3.3)^32^; all data processing was performed on-the-fly using the Appion software suite (version 3.3)^28^. Particles were extracted from dose-weighted, aligned micrographs, and analyzed using Relion-3 (version 3.0.8)^33^ for 2D classification and cryoSPARC (version 2)^34^ for 3D classification and refinement. Resolution values are according to the 0.143 gold standard Fourier shell correlation (GSFSC) method^35^.

### CKB-ASB9-ELOB-ELOC cryo-EM structure determination

Multiple datasets were collected on grids prepared with varying protein (5–20 µM) and detergent (0.01–0.1% w/v β-OG) concentrations. All datasets were collected using the same exposure rate, total exposure, frame rate, and magnification, and then merged and processed as a single dataset. In total, 1983 movies were collected and 1735 remained after removing micrographs with crystalline ice or ethane contamination. An initial dataset of 1,309,964 particles was subjected to multiple rounds of 2D classification in Relion-3 (version 3.0.8), with particles re-centered and re-extracted after each round. This yielded a final dataset of 368,831 “clean” particles. An ab initio 3D model was determined in cryoSPARC v2, asking for three initial models. The best model had clear secondary structure and was composed of 285,155 particles. A final refinement was performed using non-uniform refinement in cryoSPARC v2, yielding a final reconstruction at a resolution of ~4.1 Å. Local resolution estimation was performed in cryoSPARC v2, showing a range from 3.5 to 8 Å resolution, with the bulk of the model below 4.0 Å. A sharpened map was used for model building in Rosetta (version 3.7). Sharpened, unfiltered, and half maps were deposited in the EMDB and EMD-21120.

Our cryo-EM map showed clear density for a CKB dimer (3DRB.pdb) and the ASB9-ELOB/C heterotrimer (3ZKJ.pdb). To make an initial model, both crystal structures were docked into the cryo-EM map using Chimera version 1.13.1rc^36^. The CKB dimer seemed to be in the “open” conformation, corresponding to chain A of 3DRB. An initial model was made using chain A of PDB 3DRB to make an “open” CKB dimer which was fitted into the CKB density. Chains D, E, F of the ASB9-ELOB/C structure (PDB 3ZKJ) fit into our cryo-EM map of this part of the complex without any major conformational changes, except the N-terminal region of ASB9 needed to be modeled because residues 1–34 of ASB9 were not defined in the 3ZKJ crystal structure. The CKB-ASB9-ELOB/C model was then improved using Rosetta sub-programs RosettaCM and Rosetta *FastRelax* integrated into a cloud-based cryo-EM pipeline^37–39^. First, our starting model was used to generate ~1000 models using RosettaCM. Then the models were sorted by Rosetta energy score, and the top 10% were then scored using MolProbity (implemented in Phenix version 1.13). The 10 models with the best MolProbity score were selected as our top models. To build the N-terminal loop of ASB9 for which clear density was observed, a small region of ASB9 and CKB were extracted and used as an initial model for RosettaCM. No model was given for the ASB9 N-terminus (residues 25–35), and Rosetta CM was allowed to build this region de novo. ~500 Models were generated. This initial building yielded a large range of realistic and unrealistic structures, and 10 models with good fit to the density were selected as starting models for another round of RosettaCM. ~1000 Models were generated in this analysis, and the top 10 models as judged by Rosetta energy score and MolProbity score had good agreement (per-residue RMSD < 2 Å). The top 10 models from the two analyses were used to make 10 chimeric models that were used as inputs for Rosetta *FastRelax*, which refines side chain placement and per-residue *B*-factors. This refinement improved the MolProbity and ClashScore for each model. A final model consisting of 10 models was deposited as 6VPH.pdb.

### CUL5-RBX2 cryo-EM structure determination

Initial small datasets (~50 micrographs) showed distinct particles with good distribution in the ice, but 2D classification showed a nearly complete preferred orientation and most particles segregated into classes representing a single view. ~20,000 Particles were used for ab initio model generation and 3D refinement in cryoSPARC v2, yielding a highly anisotropic map with better than 5 Å resolution in a single dimension. To increase the angular distribution of particles in our dataset, we supplemented protein samples with the detergent n-octyl-β-d-glucopyranoside (β-OG). Multiple datasets were collected on grids prepared with varying protein (1–5 µM) and detergent (0.01–0.05% w/v β-OG) concentrations. Unfortunately, the addition of detergent did not significantly increase the angular distribution of the particle dataset. To overcome the preferred orientation in our grids, we collected data with a 20° tilt angle. Per-particle defocus was determined using gctf through the Appion wrapper. Particles were extracted and subjected to 2D classification. A total of 591 micrographs were collected and 415,601 particles were extracted for analysis. Particles were subjected to multiple rounds of 2D classification in Relion 3 and particles were re-centered and re-extracted after each round of classification. After “cleaning” the dataset in Relion 3, 44,372 unbinned particles were re-extracted with a box size of 200 and moved to cryoSPARC for ab initio model generation and 3D refinement. The best map was obtained using non-uniform (NU) refinement, yielding a final re-construction with an overall GSFSC of 5.2 Å. Analysis using the 3D Fourier Shell Correlation (3DFSC) server (www.3DFSC.salk.edu) showed that our final reconstruction has a sphericity of 0.787, improved from a sphericity of 0.646 for untilted data (sphericity of 1.000 represents a perfect particle distribution). Sharpened, unfiltered, and half maps were deposited in the EMDB as EMD-21121.

Our cryo-EM map shows clear density for the full CUL5-RBX2 complex and allows for unambiguous docking of crystal structures containing fragments of CUL5 and RBX2. To make an initial model, we used USCF Chimera to dock in crystal structures of CUL5_401-780_-RBX1 (3DPL.pdb) and the CUL5 portion of CUL5_10-387_-ELOB/C-SOCS2 (4JGH.pdb; chain D). RBX1 was mutated and re-numbered to match the highly homologous RBX2 (>50% sequence identity). RBX2 binds to three zinc molecules, and only 2 of the residues important for binding had differences between the two sequences (RBX1 His82 becomes RBX2 Cys87; RBX1 Asp97 becomes RBX2 Cys102). The only insertion/deletion was in a disordered loop. After docking each crystal structure, regions of the new homology model were docked individually (CUL5_11–150_; CUL5_696–780_; RBX2_27–44_ + CUL5_520–690_; RBX2_45–113_). The central region of CUL5, residues 165–515, showed movement of individual helices relative to the crystal structure and could not easily be docked as a rigid body. Instead of docking individually helices, ISOLDE^40^ was used in UCSF ChimeraX (version 0.91) to flexibly fit the entire region, including loops and helices. The full model was then subjected to one round of Rosetta Relax to improve packing between regions that had been docked individually. Because RBX2 coordinates several metal ions, RBX2_45–113_ was exempted from this refinement to preserve the crystal structure coordinates of 3DPL.pdb. The final model has a ClashScore of 13.63 and a MolProbity score of 2.09. Side chains were trimmed to the beta carbon position as elucidation of side chain rotamers is precluded by the moderate resolution of our map. The final coordinates were deposited in the PDB as 6V9I.pdb.

### Homology modeling

A model of the full ASB9-CRL was prepared by superimposing homologous domains of known structures to position each component in PyMOL (version 2.3.0). The CKB-ASB9-ELOB/C structure was determined as described above. ELOB-ELOC in SOCS2-ELOB/C-CUL5_NTD_(4JGH) were aligned with CKB-ASB9-ELOB-ELOC in PyMOL, and SOCS2-ELOB/C from 4JGH were removed. To orient the CUL5_CTD_ relative to CUL5_CTD_, CUL2 from CUL2-RBX1-ELOB/C-VHL (5N4W) was aligned to residues 306–387 on CUL5_NTD_, and CUL5_CTD_ from CUL5_CTD_-RBX1 (3DPL) was aligned to residues 383–424 on CUL2. After orienting the two halves of CUL5, CUL2-RBX1-ELOB/C-VHL was removed from the model. Residues 208–238 of RNF4 in RNF4-UBE2D1-Ub (4AP4) were aligned to RBX1 to position UBE2D1-Ub, and RNF4 was removed. CDC34 (6NYO) was aligned with E2D1, and E2D1 was removed to yield a model containing CDC34.

Homology modeling with MODELLER (version 9.23) was performed for each of these superimposed complexes to refine loops, fill sequence gaps, correct mutations, and to model RBX2 by sequence homology to RBX1. Sequences extracted from the PDBs were aligned to the full-length Human sequences for each protein using the pairwise alignment tool EMBOSS Water from EMBL-EBI. An alignment file for MODELLER^41^ was generated using the sequence alignments. MODELLER 9.23 was run using the default automodel class, to generate 10 models. The model with the lowest MOLPDF score was selected as the final model.

### Reporting summary

Further information on research design is available in the Nature Research Reporting Summary linked to this article.

### Supplementary information

Supplementary Information

Reporting Summary

### Source data

Source Data

## Data Availability

All HDX-MS data is available at Massive.ucsd.edu with the dataset identifier: MSV000084806. The structure of CKB-ASB9-ELOB/C is deposited with PDB code 6V9H and EMD code 21120, and the structure of CUL5-RBX2 is deposited with PDB code 6V9I and EMD code 21121. The source data underlying Figs. 4a–c, 5a, b, and 6 and Supplementary Figs. 5, 7, and 10 are provided as a Source Data file. Other data are available from the corresponding author upon reasonable request.
