# Author Correction: Structure and dynamics of the ASB9 CUL-RING E3 Ligase

**DOI:** 10.1038/s41467-023-43056-x

**Published:** 2023-11-17

**Authors:** Ryan J. Lumpkin, Richard W. Baker, Andres E. Leschziner, Elizabeth A. Komives

**Affiliations:** 1 Department of Chemistry and Biochemistry, University of California, San Diego, 9500 Gilman Drive, La Jolla, CA 92092-0378 USA; 2 Department of Cellular and Molecular Medicine, School of Medicine, University of California, San Diego, La Jolla, CA 92093 USA; 3 Section of Molecular Biology, Division of Biological Sciences, University of California, San Diego, La Jolla, CA 92093 USA; 4 Present Address: Department of Biochemistry and Biophysics, School of Medicine, University of North Carolina, Chapel Hill, Chapel Hill, NC USA; 5 Present Address: Lineberger Comprehensive Cancer Center, University of North Carolina, Chapel Hill, Chapel Hill, NC USA

Correction to: *Nature Communications* 10.1038/s41467-020-16499-9, published online 08 June 2020

In the original version of this Article, the Acknowledgements section included an incorrect NSF MCB grant number ‘817774’. The correct version states ‘NSF MCB 1817774’ in place of ‘NSF MCB 817774’.

This has been corrected in both the PDF and HTML versions of the Article.

