# Supplementary material for: Structure and dynamics of the ASB9 CUL-RING E3 Ligase
Source: Nat Commun. 2020 Jun 8;11:2866. doi: 10.1038/s41467-020-16499-9 (PMC7280518; doi:10.1038/s41467-020-16499-9)
Supplement: Supplementary file 1 — Supplementary Information [file 41467_2020_16499_MOESM1_ESM.pdf]

Supplementary Information for

Structure and dynamics of the ASB9 CUL-RING E3 Ligase

Ryan J. Lumpkin[1,‡], Richard W. Baker[2,4,‡], Andres E. Leschziner, and Elizabeth A. Komives*

1Department of Chemistry and Biochemistry, University of California, San Diego, 9500 Gilman Drive, La Jolla, CA 92092-0378, USA

2 Department of Cellular and Molecular Medicine, School of Medicine, University of California, San Diego, La Jolla, California 92093, USA

3 Section of Molecular Biology, Division of Biological Sciences, University of California San Diego, La Jolla, California 92093, USA

4 present address: Department of Biochemistry and Biophysics, School of Medicine, University of North Carolina, Chapel Hill, Chapel Hill, NC, USA; Lineberger Comprehensive Cancer Center, University of North Carolina, Chapel Hill, NC, USA

[‡]These two authors contributed equally

*Corresponding author: ekomives@ucsd.edu

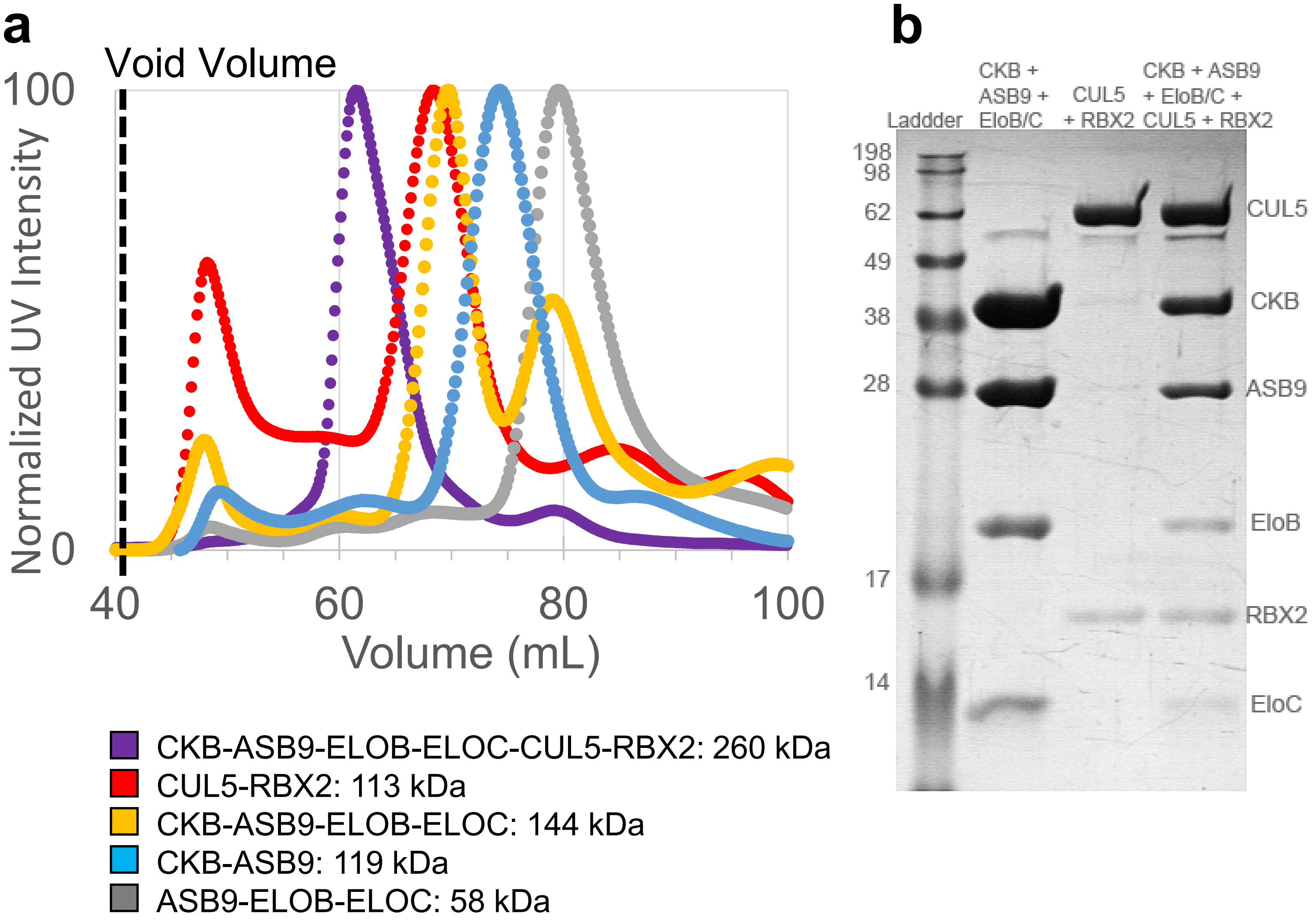

Supplementary Figure 1. Size exclusion chromatography was used to purify the various complexes studied in this work. a) Elution profiles of the various complexes b) SDS PAGE showing the purity of the complexes and the presence of the subunits present in each.

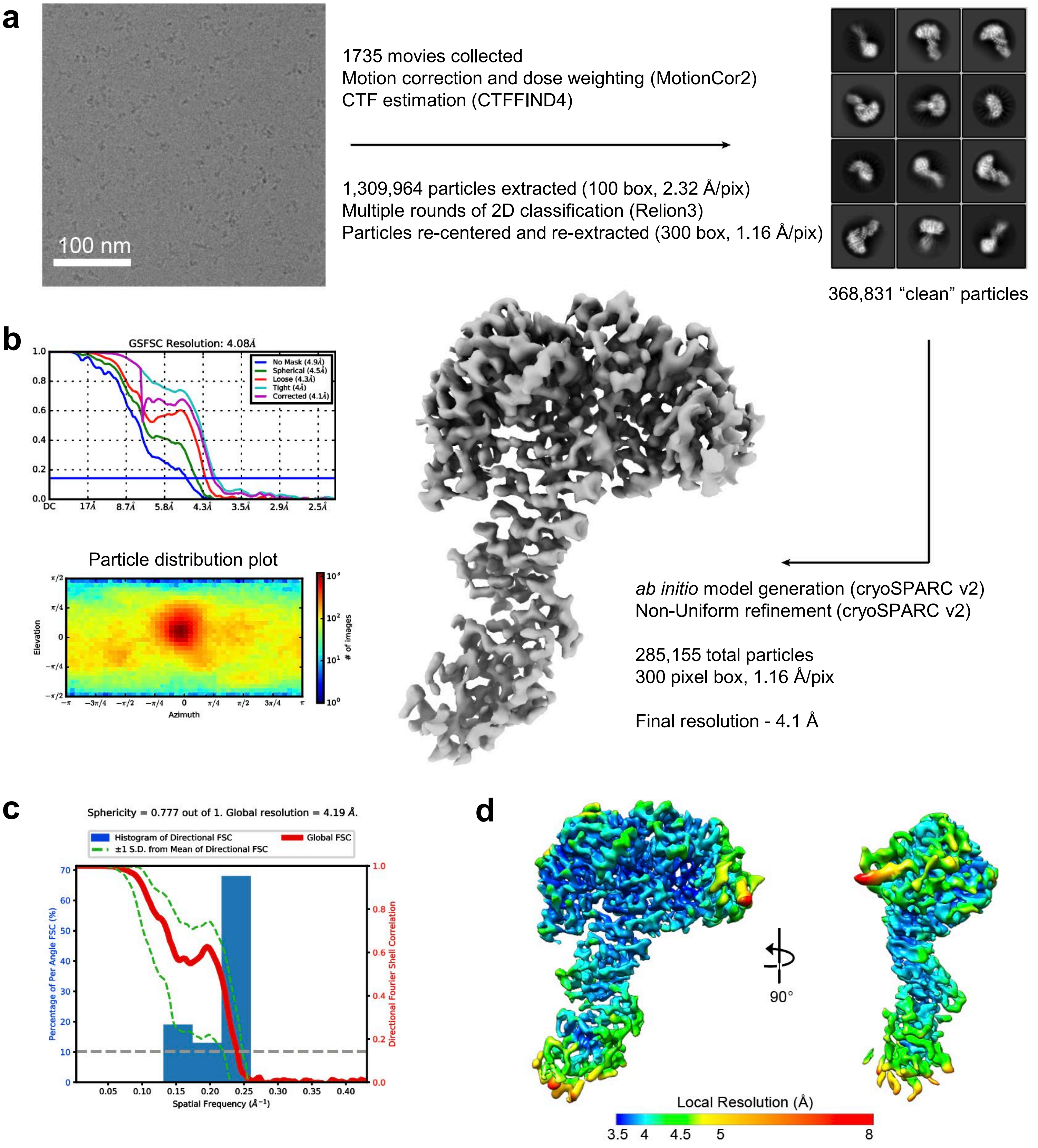

Supplementary Figure 2. cryo-EM and 2D and 3D refinement of Asb9/CKB/EloB/EloC complex (ACE)
a) Representative motion-corrected, dose-weighted cryo-EM micrograph of ACE complex (left). An overview of data processing and 2D classification (right) to yield a final “clean” particle dataset is described. 3D classification and refinement scheme is shown. A final model was refined in cryoSPARC v2, yielding a final reconstruction with a global GSFSC resolution of ~4.1 Å (map shown center).
b) FSC curves and particle distribution plot are shown
c) 3D FSC curve is shown, demonstrating that the map suffers from an anisotropic particle distribution
d) The final ACE cryo-EM map is shown colored by local resolution.

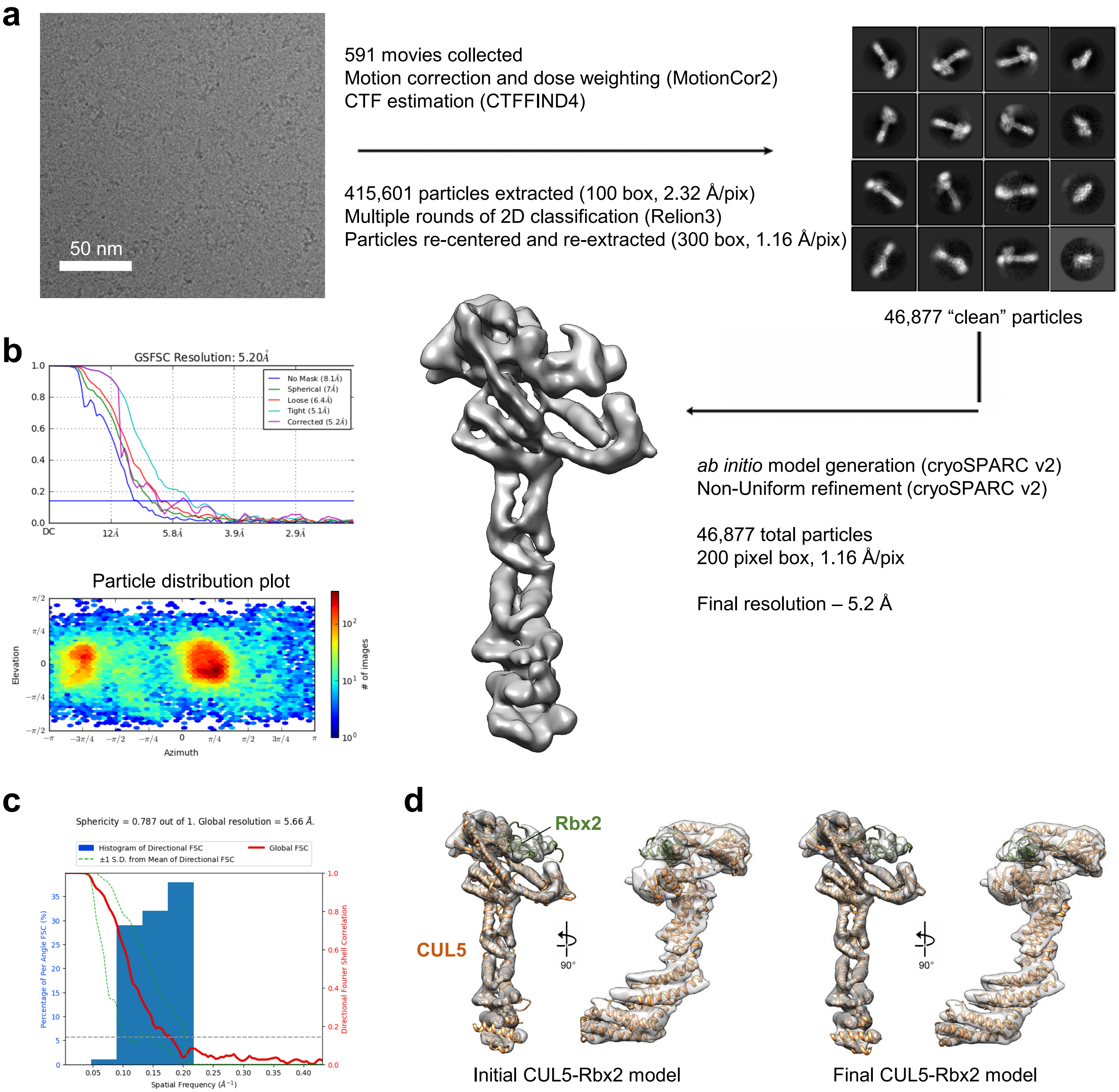

Supplementary Figure 3. cryo-EM and 2D and 3D refinement of CUL5-Rbx2 complex
a) Representative motion-corrected, dose-weighted cryo-EM micrograph of CUL5-Rbx2 complex (left). An overview of data processing and 2D classification (right) to yield a final “clean” particle dataset is described. 3D classification and refinement scheme is shown. A final model was refined in cryoSPARC v2, yielding a final reconstruction with a global GSFSC resolution of ~5.2 Å (map shown center).
b) FSC curves and particle distribution plot are shown (left).
c) 3D FSC curve is shown, demonstrating that the map suffers from an anisotropic particle distribution
d) Crystal structures (3DPL.pdb and 4JGH.pdb) containing various fragments of the CUL5-Rbx2 complex were docked into the map using UCSF Chimera (left). The final refined model is shown (right). CUL5 (orange), Rbx2 (green).

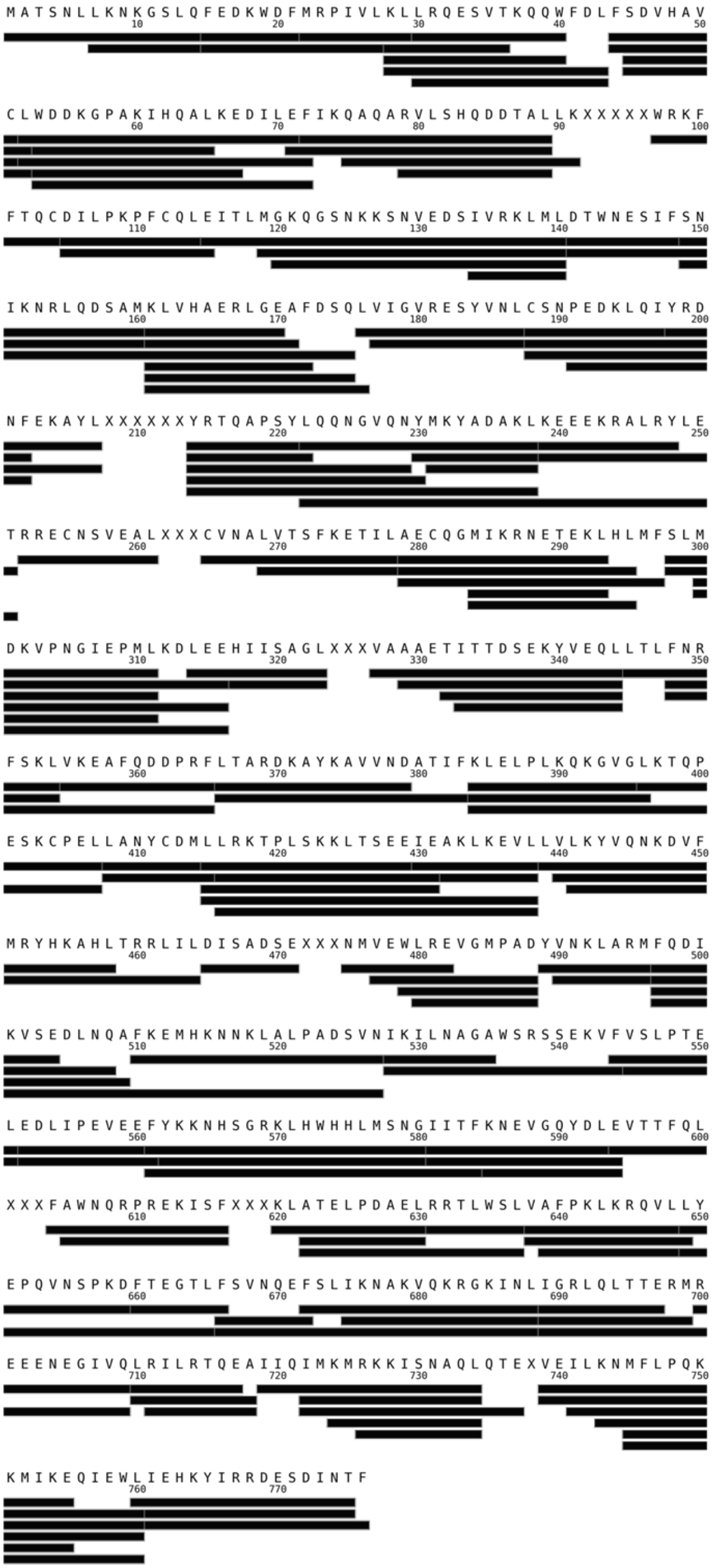

Supplementary Figure 4. Coverage map of CUL5 peptides used in the HDX-MS data analysis

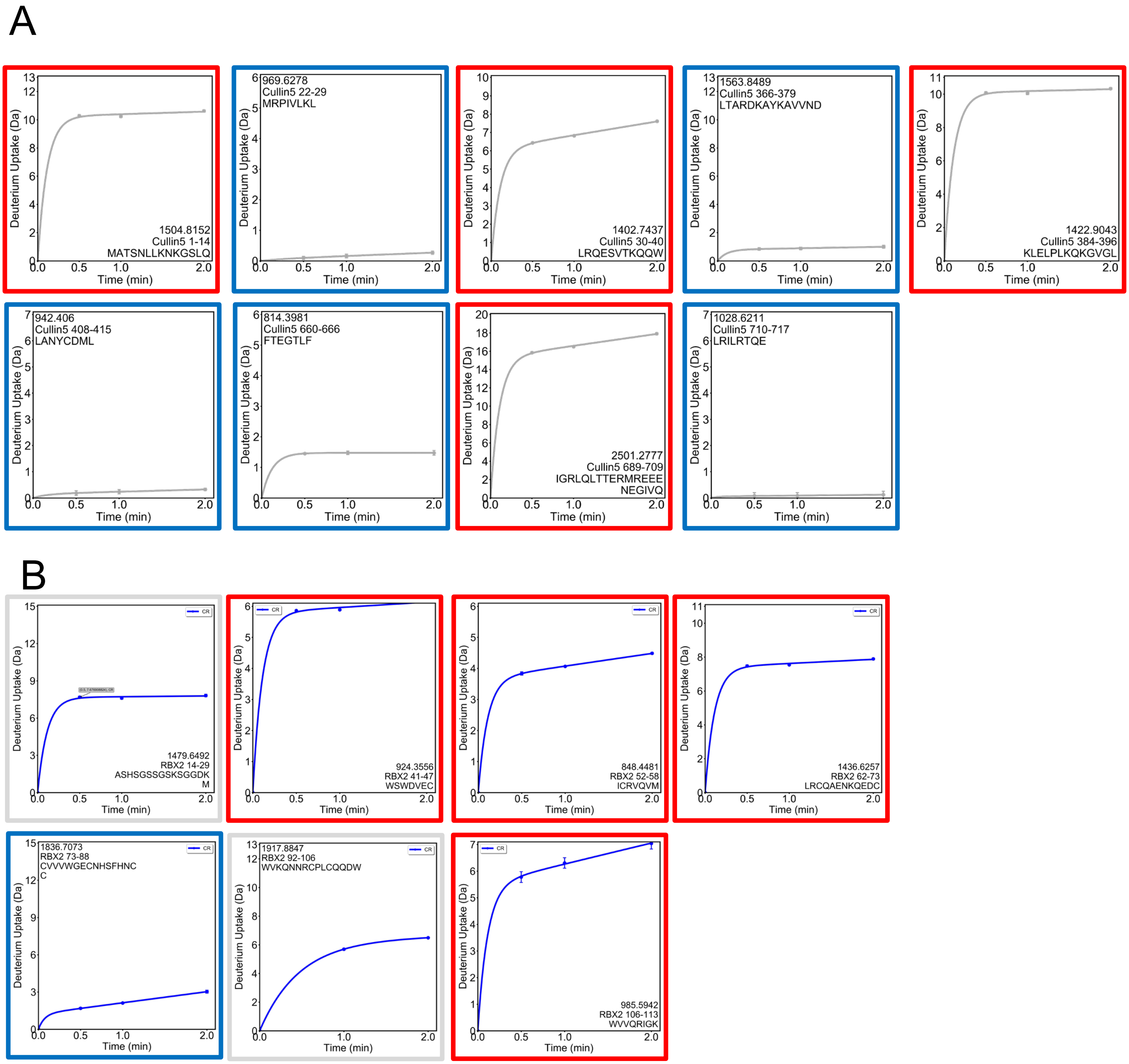

Supplementary Figure 5. A) Representative deuterium uptake plots of CUL5 peptides used in the HDX-MS data analysis presented in Figure 2B. Peptides from core regions are boxed in blue and peptides from loop regions are boxed in red. B) Representative deuterium uptake plots of RBX2 peptides used in the HDX-MS data analysis presented in Figure 2B. For all HDX-MS data, at least 2 biological replicates were analyzed each with 3 technical replicates. Data are represented as mean values +/- SEM of 3 technical replicates due to processing software limitations, however the LEAP robot provides highly reproducible data for biological replicates.

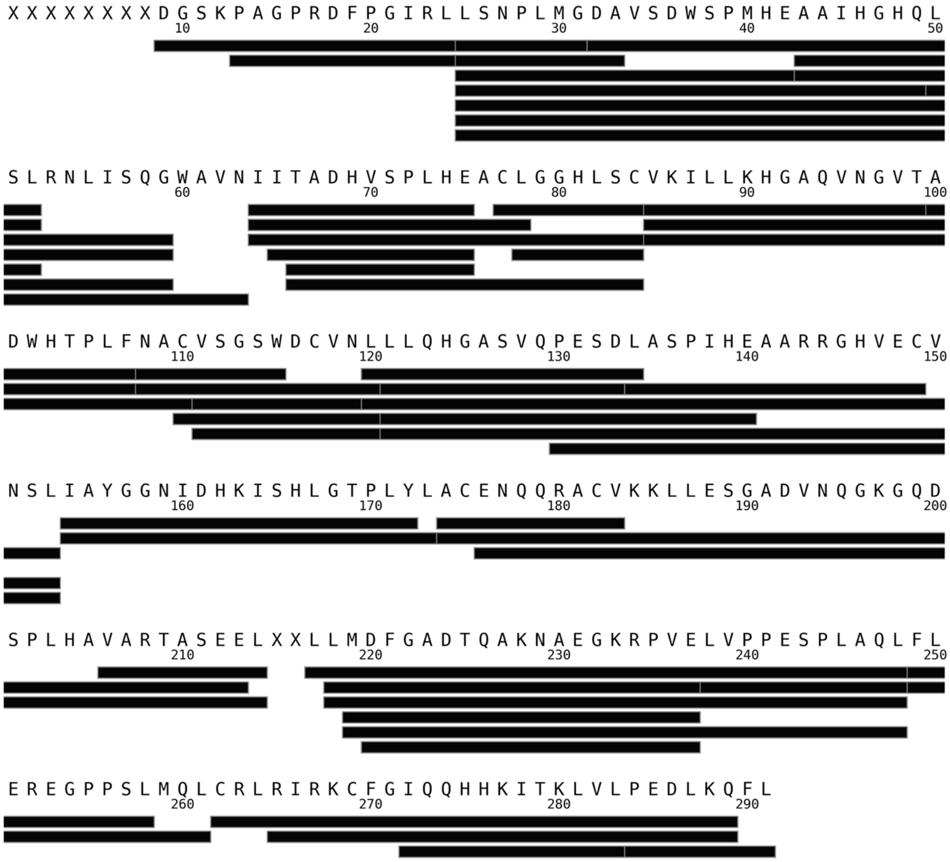

Supplementary Figure 6. Coverage map of ASB9 peptides used in the HDX-MS data analysis

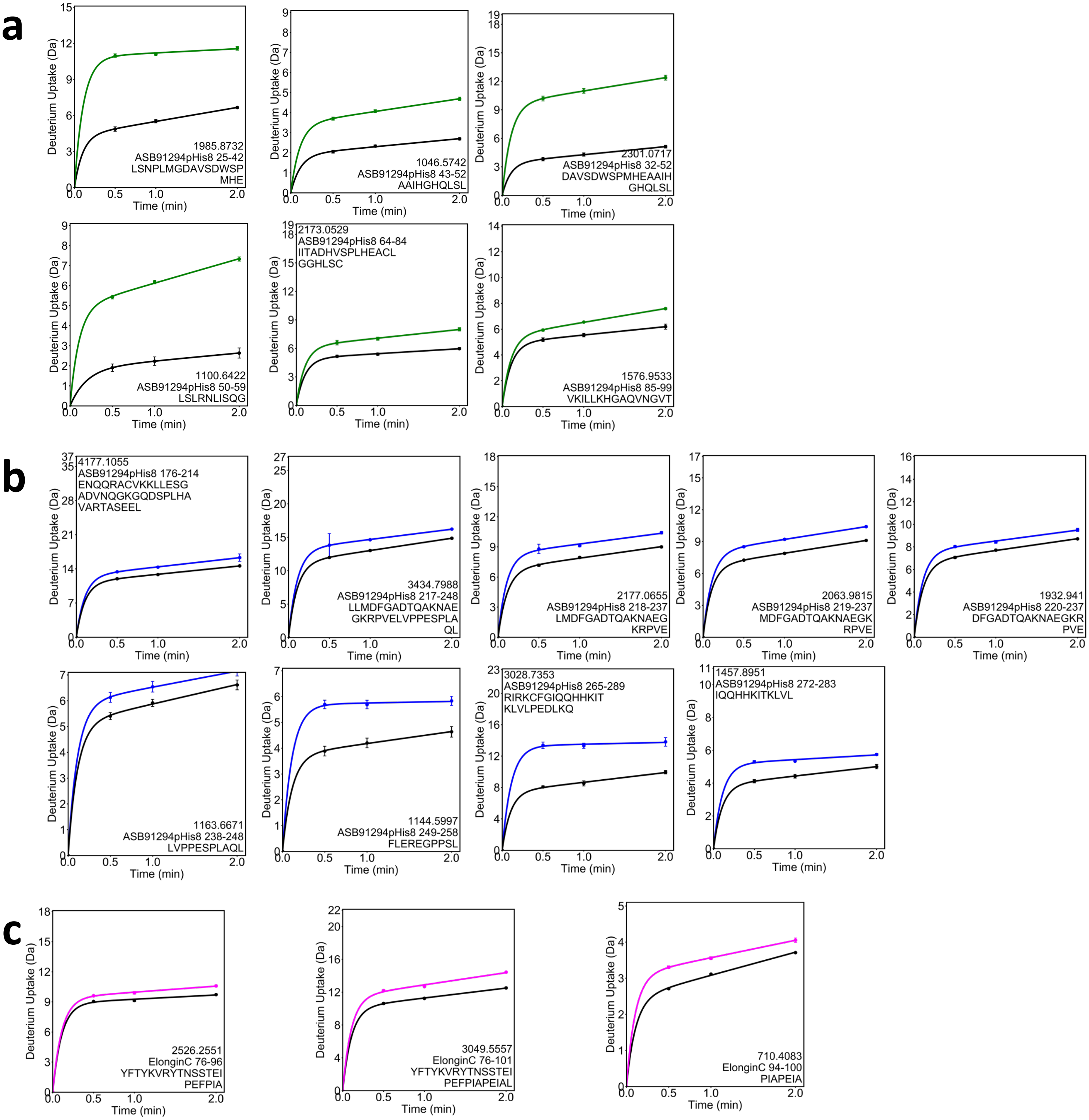

Supplementary Figure 7. Additional deuterium uptake plots for various subcomplexes. a) Deuterium uptake plots for ASB9 residues 25-31, 25-42, 43-52, 50-59, 64-84, 85-99 and 100-107 in ASB9-ELOB/C (green) vs CKB-ASB9-ELOB/C (black). b) Deuterium uptake plots for ASB9 residues 176-214, 217-248, 218-237, 219-237, 220-237, 249-258, 265-289, and 272-283 in CKB-ASB9 (blue) vs. CKB-ASB9-ELOB/C (black). c) Deuterium uptake plots for ELOC residues 76-96, 76-101, and 94-100 in CKB-ASB9-ELOB/C-CUL5-RBX2 (magenta) vs. CKB-ASB9-ELOB/C (black). For all HDX-MS data, at least 2 biological replicates were analyzed each with 3 technical replicates. Data are represented as mean values +/- SEM of 3 technical replicates due to processing software limitations, however the LEAP robot provides highly reproducible data for biological replicates. ANOVA analyses and t tests with a p value cutoff of 0.05 implemented in the program, DECA, were used to determine the significance of differences between HDX data points.

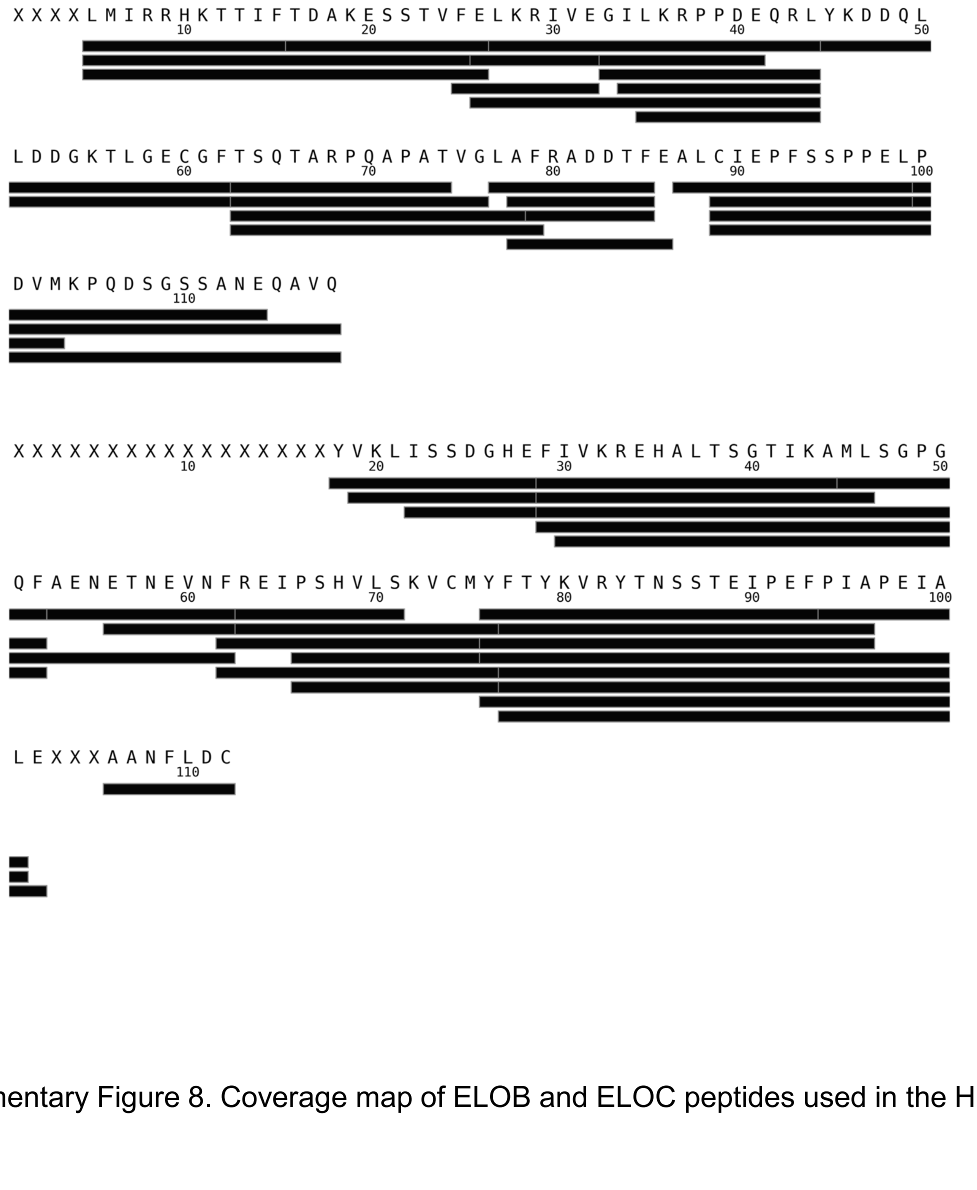

Supplementary Figure 8. Coverage map of ELOB and ELOC peptides used in the HDX-MS data analysis

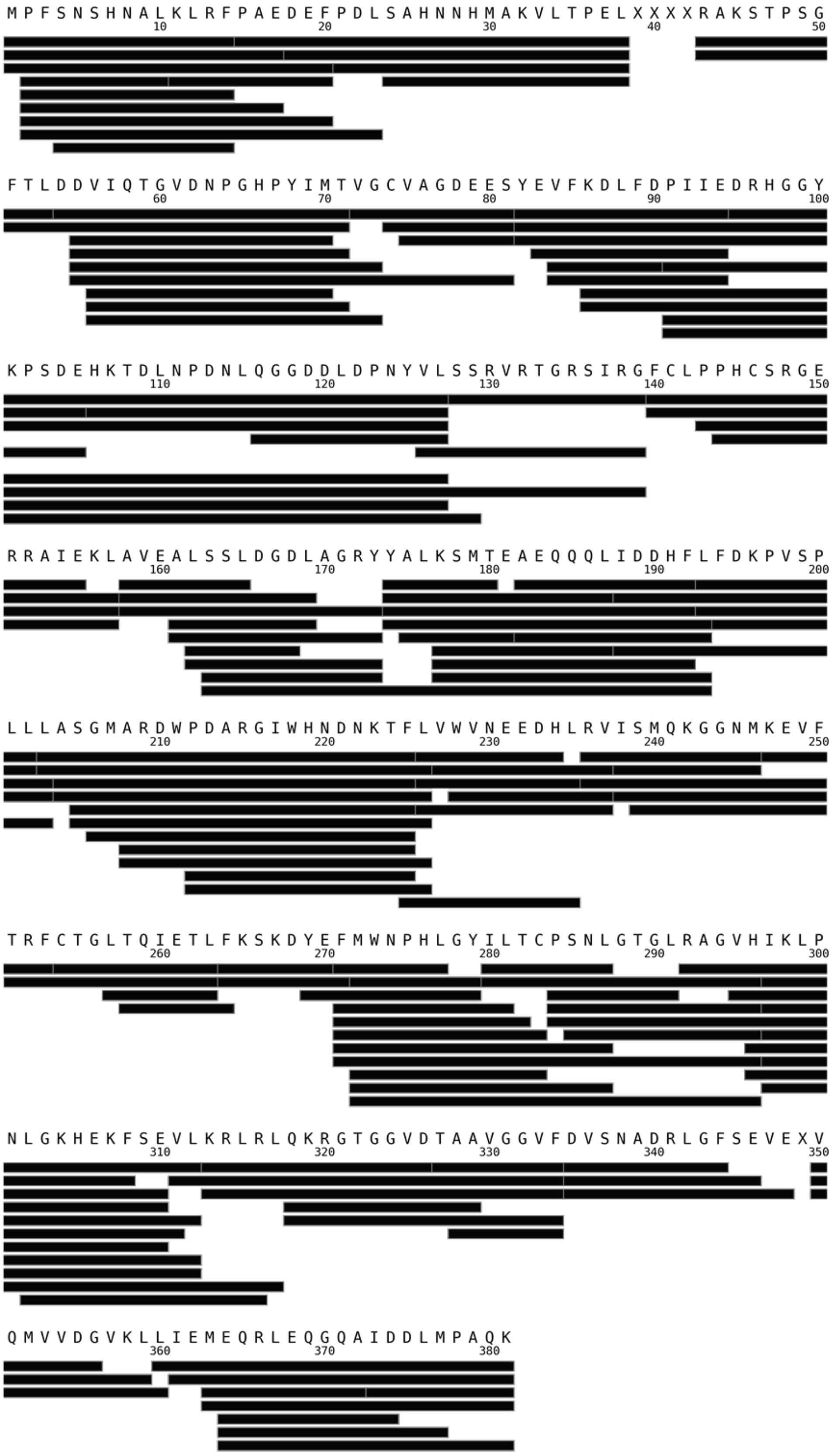

Supplementary Figure 9. Coverage map of CKB peptides used in the HDX-MS data analysis

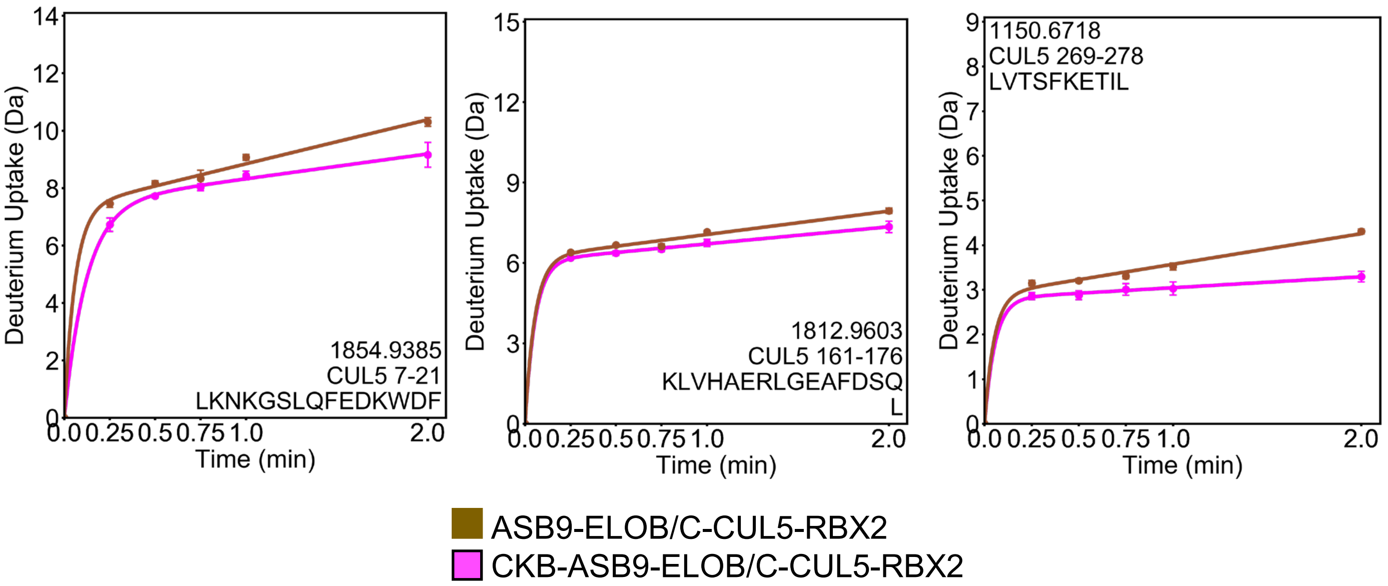

Supplementary Figure 10. Deuterium uptake plots for CUL5 residues 7-21, 161-167, 269-278. For all HDX-MS data, at least 2 biological replicates were analyzed each with 3 technical replicates. Data are represented as mean values +/- SEM of 3 technical replicates due to processing software limitations, however the LEAP robot provides highly reproducible data for biological replicates. ANOVA analyses and t tests with a p value cutoff of 0.05 implemented in the program, DECA, were used to determine the significance of differences between HDX data points.

Supplementary Table 1. Primers used in the PCR of RBX2

Gene	Original Vector	Primer (5'-3')	Destination Vector	End of gene	Restriction Site
RBX2	pRSF-Duet	CAT GCA TGC ATA TGG CGG ATG TTG AAG ATG GC	pET11a	5'	NdeI
RBX2	pRSF-Duet	CAT GCA TGG GAT CCT TAT TTG CCA ATA CGC TGC ACA AC	pET11a	3'	BamHI
