# Supplementary material for: Structure and dynamics of the ASB9 CUL-RING E3 Ligase
Source: Nat Commun. 2020 Jun 8;11:2866. doi: 10.1038/s41467-020-16499-9 (PMC7280518; doi:10.1038/s41467-020-16499-9)
Supplement: Supplementary file 2 — Reporting Summary [file 41467_2020_16499_MOESM2_ESM.pdf]

nature research

Corresponding author(s): Elizabeth A Komives

Last updated by author(s): Feb 4, 2020

Reporting Summary

Nature Research wishes to improve the reproducibility of the work that we publish. This form provides structure for consistency and transparency in reporting. For further information on Nature Research policies, see Authors & Referees and the Editorial Policy Checklist.

Statistics

For all statistical analyses, confirm that the following items are present in the figure legend, table legend, main text, or Methods section.

n/a	Confirmed	
☐	☒	The exact sample size (*n*) for each experimental group/condition, given as a discrete number and unit of measurement
☐	☒	A statement on whether measurements were taken from distinct samples or whether the same sample was measured repeatedly
☒	☐	The statistical test(s) used AND whether they are one- or two-sided<br>*Only common tests should be described solely by name; describe more complex techniques in the Methods section.*
☒	☐	A description of all covariates tested
☒	☐	A description of any assumptions or corrections, such as tests of normality and adjustment for multiple comparisons
☐	☒	A full description of the statistical parameters including central tendency (e.g. means) or other basic estimates (e.g. regression coefficient) AND variation (e.g. standard deviation) or associated estimates of uncertainty (e.g. confidence intervals)
☒	☐	For null hypothesis testing, the test statistic (e.g. *F*, *t*, *r*) with confidence intervals, effect sizes, degrees of freedom and *P* value noted<br>*Give P values as exact values whenever suitable.*
☒	☐	For Bayesian analysis, information on the choice of priors and Markov chain Monte Carlo settings
☒	☐	For hierarchical and complex designs, identification of the appropriate level for tests and full reporting of outcomes
☒	☐	Estimates of effect sizes (e.g. Cohen's *d*, Pearson's *r*), indicating how they were calculated

Our web collection on statistics for biologists contains articles on many of the points above.

Software and code

Policy information about availability of computer code

Data collection	Cryo-EM data was collected using Leginon Version 3.3, peak-picking was performed with DogPicker.py (within Appion v3.3)<br>Waters MassLynx v4.1 was used for collection of HDX-MS data.
Data analysis	Cryo-EM data was processed using Appion version 3.3, MotionCor2 v1.2.1, Relion-3 v3.0.8, CTFFIND4 v4.1.2, Relion-3 v3.0.9, and cryoSPARC v2.<br>Waters PLGS v3.0, DynamX v3.0 and DECA v110 software used for analysis, correction, and visualization of HDX-MS data.<br>Modeling was performed using MODELLER v 9.23. All Rosetta including RosettaCM, Rosetta FastRelax, Rosetta Relax, were implemented in Rosetta v3.7. MolProbity, Chimera v 1.12.1rc, ChimeraX v0.91, PyMOL v2.3.0

For manuscripts utilizing custom algorithms or software that are central to the research but not yet described in published literature, software must be made available to editors/reviewers. We strongly encourage code deposition in a community repository (e.g. GitHub). See the Nature Research guidelines for submitting code & software for further information.

Data

Policy information about availability of data

All manuscripts must include a data availability statement. This statement should provide the following information, where applicable:

- Accession codes, unique identifiers, or web links for publicly available datasets
- A list of figures that have associated raw data
- A description of any restrictions on data availability

cryo-EM data and accompanying models were deposited in the EM Data bank and Protein Data Bank as EMD-21120, 6VPH.pdb and EMD-21121, 6V9I.pdb. HDX-MS raw data has been published as a MassIVE Dataset (Accession # MSV000084806) at the Center for Computational Mass Spectrometry.

Field-specific reporting

Please select the one below that is the best fit for your research. If you are not sure, read the appropriate sections before making your selection.

☒ Life sciences ☐ Behavioural & social sciences ☐ Ecological, evolutionary & environmental sciences

For a reference copy of the document with all sections, see nature.com/documents/nr-reporting-summary-flat.pdf

Life sciences study design

All studies must disclose on these points even when the disclosure is negative.

Sample size	HDX-MS data were collected on at least two biological replicates and three technical replicates were performed for each biological replicate.
Data exclusions	HDX-MS data was excluded if peptides were not detected in at least two replicates, or if high signal-to-noise prevented accurate peptide assignment.
Replication	Every HDX-MS experiment was performed in triplicate. Biological replicates were also performed in triplicate.
Randomization	cryo-EM reconstructions use two randomized half-sets to prevent over-refinement of the model and to accurately assess the resolution of the final model.
Blinding	Blinding was implemented by use of a LEAP robot to collect the HDX-MS data

Reporting for specific materials, systems and methods

We require information from authors about some types of materials, experimental systems and methods used in many studies. Here, indicate whether each material, system or method listed is relevant to your study. If you are not sure if a list item applies to your research, read the appropriate section before selecting a response.

Materials & experimental systems

n/a	Involved in the study	
☒	☐	Antibodies
☒	☐	Eukaryotic cell lines
☒	☐	Palaeontology
☒	☐	Animals and other organisms
☒	☐	Human research participants
☒	☐	Clinical data

Methods

n/a	Involved in the study	
☒	☐	ChIP-seq
☒	☐	Flow cytometry
☒	☐	MRI-based neuroimaging
